# Stromal induction of BRD4 phosphorylation Results in Chromatin Remodeling and BET inhibitor Resistance in Colorectal Cancer

**DOI:** 10.1038/s41467-021-24687-4

**Published:** 2021-07-21

**Authors:** Wenyu Wang, Yen-An Tang, Qian Xiao, Wee Chyan Lee, Bing Cheng, Zhitong Niu, Gokce Oguz, Min Feng, Puay Leng Lee, Baojie Li, Zi-huan Yang, Yu-feng Chen, Ping Lan, Xiao-Jian Wu, Qiang Yu

**Affiliations:** 1 Guangdong Provincial Key laboratory of Colorectal and Pelvic Floor Disease, The Sixth Affiliated Hospital of Sun Yat-sen University, Guangzhou, 510655 China; 2 Guangdong Institute of Gastroenterology, The Sixth Affiliated Hospital of Sun Yat-sen University, Guangzhou, 510655 China; 3 Cancer Precision Medicine, Genome Institute of Singapore, Agency for Science, Technology, and Research, Singapore, 138672 Singapore; 4 Institute of Molecular Medicine, College of Medicine, National Cheng Kung University, Tainan, 70101 Taiwan; 5 Bio-X Institutes, Key Laboratory for the Genetics of Developmental and Neuropsychiatric Disorders, Ministry of Education, Shanghai Jiao Tong University, Shanghai, 200240 China; 6 Department of Colorectal Surgery, The Sixth Affiliated Hospital, Sun Yat-sen University, Guangzhou, 510655 China; 7 Department of Physiology, Yong Loo Lin School of Medicine, National University of Singapore, Singapore, 117597 Singapore; 8 Cancer and Stem Cell Biology, DUKE-NUS Graduate Medical School of Singapore, Singapore, 169857 Singapore

**Keywords:** Cancer therapeutic resistance, Cancer microenvironment

## Abstract

BRD4, a Bromodomain and Extraterminal (BET) protein family member, is a promising anti-cancer drug target. However, resistance to BET inhibitors targeting BRD4 is common in solid tumors. Here, we show that cancer-associated fibroblast (CAF)-activated stromal signaling, interleukin-6/8-JAK2, induces BRD4 phosphorylation at tyrosine 97/98 in colorectal cancer, resulting in BRD4 stabilization due to interaction with the deubiquitinase UCHL3. BRD4 phosphorylation at tyrosine 97/98 also displays increased binding to chromatin but reduced binding to BET inhibitors, resulting in resistance to BET inhibitors. We further show that BRD4 phosphorylation promotes interaction with STAT3 to induce chromatin remodeling through concurrent binding to enhancers and super-enhancers, supporting a tumor-promoting transcriptional program. Inhibition of IL6/IL8-JAK2 signaling abolishes BRD4 phosphorylation and sensitizes BET inhibitors in vitro and in vivo. Our study reveals a stromal mechanism for BRD4 activation and BET inhibitor resistance, which provides a rationale for developing strategies to treat CRC more effectively.

## Abstract (web summary)

BRD4 has a pro-tumorigenic role but non-cell-autonomous mechanisms of BRD4 activation need to be elucidated. Here the authors unravel a mechanism by which CAFs activate BRD4 and induce resistance to BET inhibitors in cancer cells through IL6/IL8 signaling.

## Introduction

BET family proteins, in particular, the BRD4, which are important transcriptional and epigenetic regulators, play critical roles during cancer development by modulating chromatin activity to facilitate oncogenic transcription^1^, thus elicited a great level of interest in developing BET inhibitors (BETi) for cancer treatment^2^. Currently, multiple BETi has shown the promise to induce cancer cell apoptosis and tumor regression and have entered multiple clinical trials including CRC and numerous other solid cancers. However, BET inhibitor resistance often emerges in various cancer types. In particular, BETi in solid tumors are not as effective as in hematological malignance. Moreover, although BETi is responsive in vitro it shows only a modest effect in vivo xenograft model^3^. These indicate a possible extrinsic resistance mechanism associated with tumor microenvironment (TME).

Current studies have been focusing on the cancer cell-autonomous mechanism of resistance to BETi including “adaptive kinome reprogramming”^4,5^, “transcriptional remodeling”^6,7^. Moreover, it has been shown that inactivation mutation of SPOP, an E3 ubiquitin ligase of BRD4, or dysregulation of the deubiquitinase DUB3 result in BRD4 protein stabilization causing intrinsic resistance to BETi^8–11^. Recently, several BET inhibitors such as dBET1, MZ1, which employed proteolysis-targeting chimeras (PROTACs) technology to degrade BRD4^12–14^, have been developed to conquer BET resistance caused by BRD4 stabilization. However, resistance to BET-PROTACs has also been reported^15^, although the mechanism need to be further investigated. Of note, there are several key residues of BRD4 in the binding pocket of JQ1 including Tyrosine 97 which are important for JQ1 to inhibit BRD4 activity^16,17^. The modification of these residues may change the binding affinity of BETi to BRD4, which results in treatment failure with current BET inhibitors including BET-PROTACs.

Pro-inflammatory factors secreted from the tumor microenvironment are important hallmarks of cancer^18^, which contributes to almost every aspect of tumorigenesis, metastasis and therapeutic resistance^19–22^. Although pro-inflammatory factors are well known to promote tumorigenesis via activating vital signaling pathways, it is less investigated whether and how they crosstalk with cancer epigenetic landscape including chromatin modulation to shape the biological behaviors of the tumor. Here, we sought to examine the role of TME-secreted inflammatory factors in the epigenetic regulation of CRC. Using patient-derived CRC cells, we profiled the chromatin regulators of CRC induced by inflammatory cytokines and identified bromodomain protein BRD4 as a key epigenetic modulator which can be activated through a paracrine IL6- and IL8-mediated JAK2 signaling. We further show the TME-induced BRD4 activation provides a previously unrecognized mechanism for chromatin remodeling and resistance to BRD4 inhibitors. Our study provides insights into TME regulation of tumor activity and a combination strategy to overcome therapy resistance.

## Results

### Pro-inflammatory cytokines interleukin 6 and interleukin 8 induce BRD4 protein expression in cancer cells

**Fig. 1:**
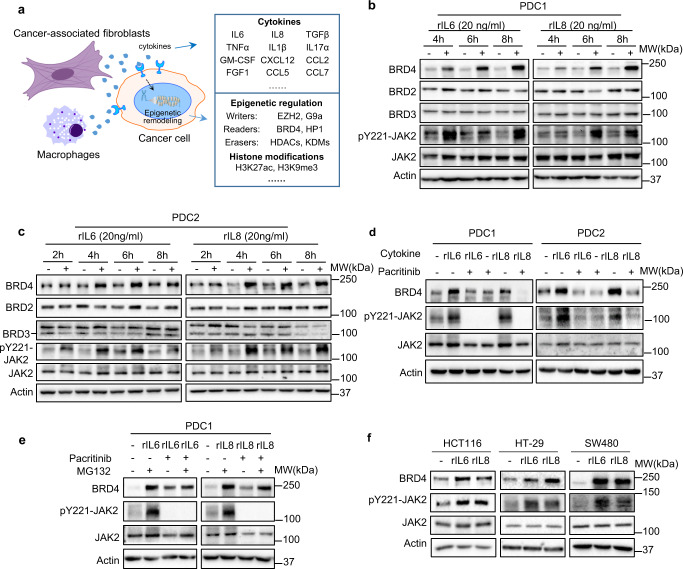
IL6 and IL8 induce BRD4 protein expression in CRC. **a** Illustration depicting a screening strategy to investigate the tumor microenvironment-derived cytokines and their proposed function on epigenetic remodeling in CRC. **b**, **c** Representative western blot analysis of indicated proteins in patient-derived cancer cells PDC1 (**b**, *n* = 3) and PDC2 (**c**, *n* = 2) after treatment with 20 ng/ml rIL6 or 20 ng/ml rIL8 for indicated time. **d** Representative western blot analysis (*n* = 2) of indicated proteins in patient-derived cancer cells treated with/without 20 ng/ml rIL6 (20 ng/ml), 20 ng/ml rIL8, or 2.5 μM pacritinib. **e** Representative western blot analysis (*n* = 2) of indicated proteins in patient-derived cancer cells treated with/without 20 ng/ml rIL6, 20 ng/ml rIL8, 2.5 μM pacritinib, or 20 μM MG132. **f** Representative western blot analysis (*n* = 2) of indicated proteins in colon cancer cell lines (HCT116, HT-29, SW480) treated with 5 ng/ml rIL6 or rIL8.

To investigate the effect of the inflammatory cytokines on chromatin activity in CRC, we treated the patient-derived colorectal cancer cell line (PDC1) with a panel of 14 recombinant cytokines, which are well-known to be involved in the inflammatory tumor microenvironment (Fig. 1a). The protein levels of major epigenetic regulators, including histone readers, writers, and erasers and associated histone makers, were analyzed by immunoblotting (Figs. 1a and S1a). Among them, we found that the recombinant interleukin 6 (rIL6) and interleukin 8 (rIL8) markedly induced the expression of histone reader protein BRD4, but did not affect the other BET family members BRD2 and BRD3 nor the other chromatin regulators (Fig. S1a). A further time course analysis using two patient-derived CRC lines (PDC1 and PDC2) confirmed that the rIL6 or rIL8 could induce BRD4 expression as early as 4 h, without affecting BRD2 and BRD3 (Fig. 1b, c). Of note, Janus kinase 2 (JAK2), which is the downstream kinase of IL6/8, was activated as early as 2 h prior to the BRD4 induction (Fig. 1c), raising a possibility that JAK2 might be a potential upstream kinase responsible for BRD4 induction. In alignment with this hypothesis, co-treatment with a JAK2 inhibitor pacritinib abolished the rIL6/8-induced BRD4 expression (Fig. 1d), but this was rescued by proteasome inhibitor MG132 (Fig. 1e). This finding indicates that increased BRD4 expression is associated with increased protein stability. Indeed, we found that rIL6 or rIL8 treatment did not increase *BRD4* mRNA levels (Fig. S1b). The rIL6/8-induced BRD4 protein expression was also confirmed in several commercial colorectal cancer cell lines (Fig. 1f). Interestingly, it was also seen in cell lines of other cancer types, including breast, lung, and prostate (Fig. S1c). These findings indicate that IL6/8-induction of BRD4 is common and well conserved in human cancers.

### JAK2 induces BRD4 protein stabilization through phosphorylation at tyrosine 97 and tyrosine 98

**Fig. 2:**
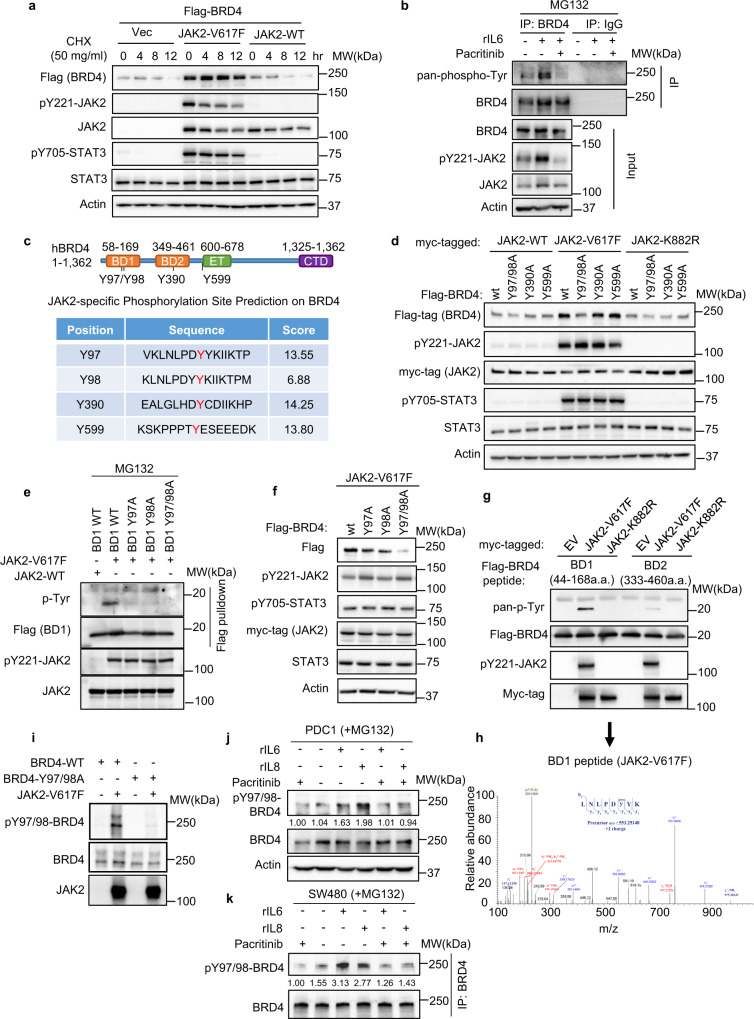
JAK2 induces tyrosine phosphorylation of BRD4 at Y97/Y98, resulting in BRD4 stabilization. **a** Representative western blot analysis (*n* = 2) of indicated proteins in 293T cells transfected with indicated constructs, treated with 50 μg/ml cycloheximide (CHX) and harvested at different time points. **b** Representative western blot analysis (*n* = 3) of whole-cell lysate (WCL) and samples from co-IP with anti-BRD4 antibody in PDC1 cells treated with 20 ng/ml rIL6 or 2.5 μM pacritinib as indicated. **c** Scheme depicting functional domains of BRD4 and prediction of BRD4 phosphorylation sites by JAK2. The tyrosine residues predictively phosphorylated by JAK2 were highlighted in red. **d** Representative western blot analysis (*n* = 3) of indicated proteins in 293T cells transfected to express the indicated BRD4 mutants together with wild-type JAK2, active JAK2-V617F, or inactive JAK2-K882R. **e** Representative western blot analysis (*n* = 2) of tyrosine phosphorylation on BRD4-BD1 and its mutants in 293T cells transfected to express BRD4-BD1 and indicated BRD4-BD1 mutants together with wild-type JAK2 or active JAK2-V617F. **f** Representative western blot analysis (*n* = 3) of indicated proteins in 293T cells transfected with BRD4 and its mutants together with active JAK2-V617F. **g** Representative western blot analysis (*n* = 2) of phosphorylation of BRD4 peptides (BD1 and BD2) from in vitro kinase assay by active JAK2-V617F or inactive JAK2-K882R. **h** Mass spectrum analysis of phosphorylated residues of BRD4 peptide by active JAK2-V617F. **i** Representative western blot analysis (*n* = 3) of anti-Flag immunoprecipitates derived from 293T cells transfected with the indicated constructs followed by incubation with active JAK2-V617F in in vitro kinase assay. **j** Representative western blot analysis (*n* = 2) of WCL from PDC1 cells treated with indicated cytokines, pacritinib, and 20 μM MG132. **k** Representative western blot analysis (*n* = 2) of immunoprecipitates by anti-BRD4 antibody from SW480 cells treated with indicated cytokines, pacritinib, and 20 μM MG132.

To validate the BRD4 stabilization by JAK2, we transfected 293T cells with a flag-tagged BRD4, together with a wild-type JAK2 (JAK2-WT) or a constitutively activated JAK2 (JAK2-V617F) and examined the BRD4 protein turnover in the presence of protein synthesis inhibitor cycloheximide. The result showed that the active JAK2-V617F, but not the JAK2-WT, induced a remarkable induction of flag-BRD4 (Fig. 2a). Upon cycloheximide treatment, JAK2-V617F-induced BRD4 retained its levels over time, indicating a prolonged half-life, while the BRD4 in mock or JAK2-WT-transfected cells showed rapid degradation (Figs. 2a and S2A). We further confirmed that JAK2-V617F but not the kinase-dead JAK2 (JAK2-K882R), was also able to induce the endogenous BRD4 (Fig. S2b).

To investigate whether BRD4 can be phosphorylated by tyrosine kinase JAK2, we detected tyrosine phosphorylation on BRD4 after immunoprecipitation. Immunoprecipitated BRD4 exhibited increased tyrosine phosphorylation in cells treated with rIL6, but not in cells co-treated with JAK2 inhibitor pacritinib (Fig. 2b), supporting that BRD4 is phosphorylated by IL6-JAK2 signaling. To predict the potential tyrosine phosphorylation sites on BRD4 by JAK2, we employed the Group-based Prediction System^23^, which lead to the prediction of four tyrosine residues (Y97, Y98, Y390, and Y599) that were potentially phosphorylatable by JAK2 (Fig. 2c). Notably, Y97/98 and Y390 are located within the bromodomains BD1 and BD2, respectively, which are known to be essential for the epigenetic reader activity of BRD4^16,17^. Mutagenesis analysis through tyrosine to non-phosphorylated alanine substitutions (Y to A) found that Y97/98 in BD1, but not Y390 or Y599, was required for JAK2-induced BRD4 stabilization (Figs. 2d and S2c), and adding proteasome inhibitor MG132 restored the stability of BRD4-Y97/98A mutant (Fig. S2d). Further dissection of Y97 and Y98 individually indicated that both tyrosine residues were required for JAK2-induced BRD4 phosphorylation (Fig. 2e) and stabilization (Fig. 2f). Interestingly, Y97 but not Y98 was found to be conserved among BET family members including BRD2 and BRD3 (Fig. S2e). This may explain that only BRD4 could be stabilized by active JAK2. Moreover, the Y97/98A mutant showed much higher levels of ubiquitination compared to other mutants (Fig. S2f). By contrast, the tyrosine-to-glutamic acid (Y to E) mutant Y97/98E that mimics constitutive phosphorylation of tyrosine residues showed an increased protein level, which was compatible with the wild-type BRD4 induced by active JAK2 (Fig. S2g). Consistently, the phosphor-mimic Y97/98E mutant showed a much lower level of ubiquitination when compared with the Y97/98A mutant (Fig. S2h). Together, these data stressed that the JAK2-induced phosphorylation of BRD4 at Y97/98 determined the stabilization of BRD4.

To confirm that BRD4 is a direct substrate of JAK2, we next performed the in vitro kinase assay. We show that the active JAK2 (V617F) but not the kinase-dead JAK2 (K882R) can induce strong tyrosine phosphorylation of the synthetic peptide of BRD4-BD1, but slight phosphorylation of BRD4-BD2 (Fig. 2g). Mass-Spectrum analysis of the phosphorylated BD1 domain peptide confirms the abundant phosphorylation at Y97, though the Y98 phosphorylation was modest (Fig. 2h). We then developed a polyclonal antibody raised specifically against the Y97/98 phosphorylated peptide of BRD4 (Fig. S3a), and confirmed that the phosphor-Y97/98 BRD4 antibody recognized specifically the JAK2-induced BD1 domain phosphorylation in the in vitro kinase assay (Fig. S3b, c). The phosphor-Y97/98 antibody also recognized the wild-type BRD4 but not the BRD4-Y97/98A mutant (Figs. 2i and S3d); it also detected the phosphorylation of endogenous BRD4 in cells treated rIL6 and rIL8, which was diminished by pacritinib (Fig. 2j, k). Taken together, we concluded that the BRD4 is a bona fide substrate of JAK2, and the phosphorylation at tyrosine 97/98 is critical for the stabilization of BRD4.

### UCHL3 deubiquitinase interacts with and stabilizes phosphorylated BRD4

**Fig. 3:**
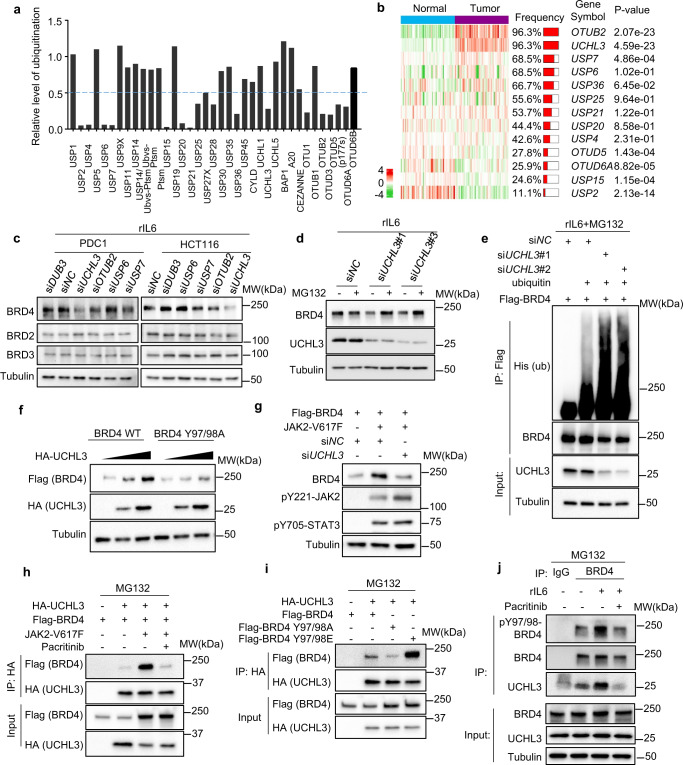
Deubiquitinase UCHL3 is required for JAK2-induced BRD4 stabilization. **a** Identification of deubiquitinases of BRD4. Purified unbiquitinated BRD4 was incubated with indicated deubiquitinases according to the manufacturer’s instructions (DUB Scan kit, Cat. No. 67-0006-001). After that, western blot analysis was performed to probe ubiquitination of BRD4 (Fig. S4a). Quantification of relative intensity of ubiquitin of BRD4 normalized to BRD4 incubated with buffer. **b** Heat map showing frequency of high expression of candidate DUBs in 50 paired CRC and adjacent normal mucosa tissues. **c** Representative western blot analysis (*n* = 2) of indicated proteins in PDC1 and HCT116 cells transfected with indicated siRNAs with 50 ng/ml rIL6. **d** Representative western blot analysis (*n* = 2) of indicated proteins in PDC1 cells transfected with indicated siRNAs with/without 20 μg/ml MG132 under rIL6 (50 ng/ml) treatment. **e** In vitro ubiquitination assays (*n* = 3) of WCL and immunoprecipitates from 293T cells transfected with indicated siRNA or constructs in the presence of 20 μM MG132 and 50 ng/ml rIL6. **f** Representative western blot analysis (*n* = 3) of WCL from 293T cell transfected with Flag-BRD4 or Flag-BRD4-Y97/98A together with HA-UCHL3 constructs. **g** Representative western blot analysis (*n* = 2) of indicated proteins in 293T cells transfected with indicated siRNAs or constructs. **h** Representative western blot analysis (*n* = 2) of WCL and immunoprecipitates by anti-HA antibody from 293T cells transfected with indicated constructs with 20 μM MG132 or 2.5 μM pacritinib as indicated. **i** Representative western blot analysis (*n* = 2) of WCL and immunoprecipitates by anti-HA antibody from 293T cells transfected with indicated constructs with 20 μM MG132. **j** Representative western blot analysis (*n* = 2) of WCL and immunoprecipitates by anti-BRD4 antibody from PDC1 cells treated with 50 ng/ml rIL6 and/or 2.5 μM pacritinib as indicated.

Having shown that increased BRD4 stabilization is associated with reduced ubiquitination, we set out to investigate the de-ubiquitination events to enable JAK2-mediated BRD4 stabilization. To this end, we performed a deubiquitinase (DUB) library screening in which ubiquitinated BRD4 as a substrate was incubated with a set of DUBs, followed by measurement of the ubiquitination level of BRD4 by western blotting (Fig. S4a). This experiment leads to the identification of 16 DUBs that showed marked de-ubiquitin activity towards BRD4 (Figs. 3a and S4a). To determine which of these DUBs are relevant in CRC, we compared their expression in a gene expression dataset of CRC and adjacent normal controls^24^. We found that *OTUB2*, *UCHL3*, and *USP7* are the top DUBs overexpressed in CRC as compared to their normal adjacent tissues with high ectopic expression frequency and significant *p* value (Frequency > 60%, *p* value < 0.01) (Fig. 3b). To determine their involvement in regulating BRD4, we performed knockdown of these DUBs in the presence of rIL6, together with *USP6* as a negative control and *DUB3*, which has been previously shown to regulate BRD4^8^. The result shows that *UCHL3* knockdown was most efficient in reducing BRD4 expression (Figs. 3c and S4b, c) and shortened BRD4 half-life (Fig. S4d, e), and this effect can be restored by proteasome inhibitor MG132 treatment (Figs. 3d and S4f). Consistently, ubiquitination assay showed knockdown of *UCHL3* promoted the poly-ubiquitination of BRD4 (Fig. 3e). We also performed *UCHL3* knockdown when active JAK2 was overexpressed. The induction of BRD4 by active JAK2 was significantly diminished by *UCHL3* knockdown (Fig. S4g). Whereas, neither protein level nor mRNA level of *UCHL3* itself was not affected by JAK2 activation (Fig. S4g, h). It has been recently reported that the E3 ligase SPOP induces BRD4 degradation^9–11^. Interestingly, we found that the concomitant knockdown of *SPOP* with *UCHL3* rescued the effect of *UCHL3* knockdown on BRD4 protein (Fig. S4i), indicating that UCHL3 antagonizes SPOP to maintain BRD4 stability. Conversely, ectopic expression of UCHL3 prolonged the half-life of BRD4 (Fig. S4j); It also increased the protein stability of WT BRD4 but not BRD4-Y97/98A mutant (Fig. 3f). Conversely, the *UCHL3* knockdown also abolished JAK2-induced BRD4 stabilization (Fig. 3g). Of note, our experiments were performed in the presence of IL6/8. whether other deubiquitinases are preferential in other contexts need to be further investigated.

To investigate if UCHL3 binds to BRD4, we performed reciprocal co-immunoprecipitation assays in cells transfected with BRD4, alone or with UCHL3 and JAK2-V617F. The result showed that UCHL3 indeed interacted with BRD4, which was greatly enhanced in the presence of JAK2-V617F, but inhibited by pacritinib (Figs. 3h and S4k). This indicates that UCHL3 preferentially binds to a phosphorylated BRD4. Indeed, the non-phosphorylation BRD4-Y97/98A mutant showed a reduced interaction with UCHL3, while the phosphorylation mimic BRD4-Y97/98E presented an increased interaction with UCHL3 (Figs. 3i and S4l). Further investigation also showed the endogenous BRD4 binding with UCHL3 which was enhanced by rIL6 but inhibited by pacritinib (Figs. 3j and S4m). Thus, we confirm that UCHL3 is a deubiquitinase for JAK2-phosphorylated BRD4, whose overexpression in CRC may provide a mechanism to maintain the BRD4 protein abundance in an inflammatory tumor microenvironment.

### CAF-secreted IL6/IL8 induces JAK2-BRD4 signaling to confer tumor aggressiveness

**Fig. 4:**
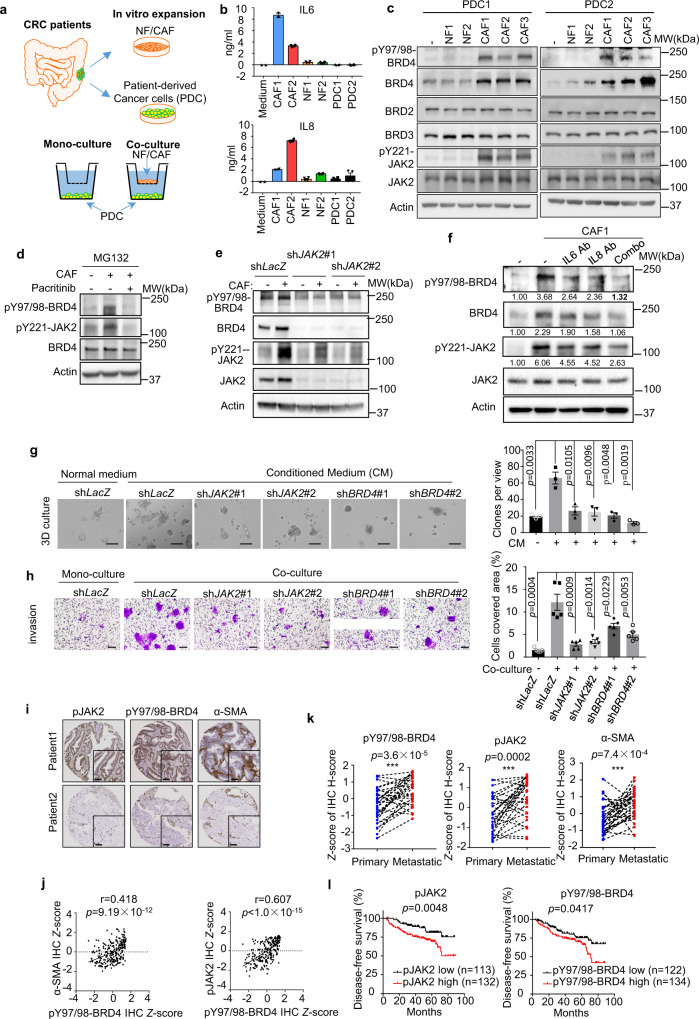
Cancer-associated fibroblasts promote phosphorylation and stabilization of BRD4, which is associated with poor outcome of CRC patients. **a** Scheme depicting the establishment of PDCs, CAFs, NFs from primary CRC samples and coculture system. **b** ELISA analysis (*n* = 4) of indicated cytokines in the culture medium where indicated cells were cultured. **c** Representative western blot analysis (*n* = 2) in patient-derived cancer cells (PDCs) after coculture with CAFs or NFs. **d** Representative western blot analysis (*n* = 2) of indicated proteins in PDC1 cells after mono-culture or coculture with CAFs. Cells were treated with 2.5 μM pacritinib, 20 μM MG132 as indicated. **e** Representative western blot analysis (*n* = 2) of indicated proteins in PDC1 cells with *JAK2* shRNAs mono-cultured or cocultured with CAFs. **f** Representative western blot analysis (*n* = 3) of indicated proteins in PDC1 cells after mono-culture/coculture with CAFs. Cells were treated with 2.5 μM IL6 antibody, IL8 antibody or combination. **g** Representative images (left panel) and quantification (right panel, *n* = 3) of cell growth in 3D Matrigel. PDC1 cells expressing indicated Tet-on-shRNAs were cultured on Matrigel with normal medium or CAF-conditioned medium supplied with 100 ng/ml doxycycline (Scale bars, 100 μm). **h** Representative images (left panel) and quantification (right panel, *n* = 5) of invasive cell in invasion assay. PDC1 cells expressing indicated Tet-on-shRNAs were mono-cultured or cocultured with CAFs with 100 ng/ml doxycycline (Scale bars, 50 μm). **i** Representative IHC staining for pJAK2, pY97/98-BRD4, and α-SMA in TMAs. H scores for each TMA core were determined by the Slidepath Tissue IA software (Leica Microsystems). (Scale bars, 100 μm). **j** Immunohistochemistry analysis of correlation between expression of pY97/98-BRD4, pJAK2 and α-SMA in CRC patients. **k** Quantification of pY97/98-BRD4, pJAK2, and α-SMA levels in IHC analyses of 32 paired primary and metastatic colorectal tumor samples. **l** Kaplan−Meier analyses of the association of disease-free survival with individual proteins (pJAK2 and pY97/98-BRD4) in 248 CRC patients (stage II and stage III) from TMAs. In **b**, **g**, and **h**, data are presented as the mean ± SEM (error bars) of at least three independent experiments or biological replicates. The *p* values were determined using two-tailed Pearson’s *χ*^2^ test in **j**, two-tailed log-rank (Mantel-Cox) test in **l**, and two-tailed Student’s *t* tests (paired in **k**, unpaired in **g** and **h**).

Cancer-associated fibroblasts (CAFs) are one of the major sources in the tumor microenvironment to secrete IL6 and IL8^25–27^. Indeed, through analyzing the colorectal cancer dataset GSE39396, we found that CAFs express higher levels of IL6 and IL8 than that in other cell types in the TME such as endothelial cells and immune cells and cancer cells^25^ (Fig. S5). We thus sought to use CAFs as a cellular model to investigate if CAFs can induce BRD4 expression in CRC via IL6/IL8 secretion. To do this, we isolated CAFs from resected tumors as well as the adjacent normal fibroblasts (NFs) from various CRC patients for CAF/CRC coculture experiments (Fig. 4a). Enzyme-linked immune sorbent assay (ELISA) analysis confirmed that the patient-derived CAFs secreted much higher levels of IL6 and IL8 compared to normal fibroblasts (NF) as well as PDC cells (Fig. 4b). Similar to rIL6/8, CAFs, but not NFs, induced BRD4 expression along with JAK2 activation in PDC1 and PDC2, but not BRD2 and BRD3 (Fig. 4c). Likewise, the treatment of the PDC cells directly with the conditioned medium of CAF (CM) also induced the BRD4 expression (Fig. 4c). CAF also yielded BRD4 Y97/98 phosphorylation, which was abolished by pacritinib (Fig. 4d), *JAK2* knockdown (Fig. 4e), or IL6 and IL8 neutralizing antibody (Fig. 4f). Of note, a simultaneous blocking of both IL6/IL8 appears to be necessary to warrant a more efficient ablation of BRD4 induction (Fig. 4f).

We further show that CAF can promote PDC growth, invasion, and migration, which can be abolished by knockdown of *JAK2* or *BRD4* (Figs. 4g, h, and  S6a, b). Treating the PDC cells with both the IL6 receptor neutralizing antibody tocilizumab and IL8 receptor inhibitor reparixin also ablated the above phenotype, though the single-agent only had modest effects (Fig. S6c–g). Furthermore, the in vivo assay showed that co-engraftment CAF with PDC1 or HT-29 strongly promoted xenograft tumor growth compared with CRC cells alone (Fig. S6h), which was in line with immunohistochemistry (IHC) detection of higher levels of pJAK2 and pBRD4 in CAF/CRC-derived xenograft tumors (Fig. S6i).

Finally, we validated the pJAK2 correlation with BRD4 or pBRD4 in CRC clinical samples. We performed IHC analysis of α-SMA (a marker for CAF), pJAK2, and pBRD4 in a tissue microarray (TMA) consisting of 248 CRC tumors with clinical information^28^ (Fig. 4i). As expected, a higher CAF infiltration (identified by α-SMA expression) in CRC was significantly correlated with pBRD4 (Pearson’s *r* = 0.418, *P* < 1.0 × 10^−11^) (Fig. 4j). A strong positive correlation between pJAK2 and pBRD4 was also confirmed (with Pearson’s *r* = 0.607, *P* < 1.0 × 10^−15^) (Fig. 4j). Moreover, an examination of a set of paired primary CRC and metastatic CRC samples showed higher levels of pBRD4 and pJAK2, along with a higher level of CAF infiltration in metastatic tumors compared to primary tumors (Fig. 4k). Disease-free survival (DFS) analysis of these 248 CRC patients reveals that the higher expressions of pJAK2 and pBRD4 were associated with poor survival outcomes in CRC patients (Fig. 4l). These data support that CAF-mediated JAK2-BRD4 activation in tumor microenvironment plays an important role in CRC progression.

### CAFs confer resistance to BET inhibitors through the IL6/8-JAK2-BRD4 cascade

**Fig. 5:**
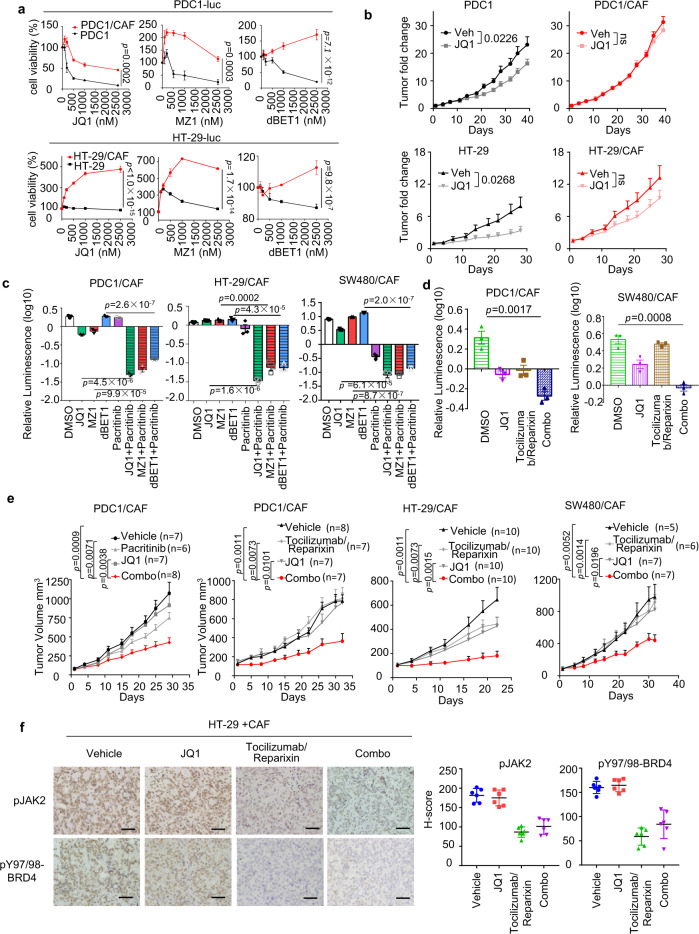
CAFs confer resistance to BET inhibitors, which can be abolished by co-inhibition of IL6/8-JAK2 signaling. **a** Cell viability of PDC1-luc cells (top, *n* = 6) and HT-29-luc cells (bottom, *n* = 3), which were mono-cultured or cocultured with CAFs, followed by treatment with different BET inhibitors with indicated concentrations. **b** NOD/SCID mice were engrafted with PDC1 or HT-29 tumor cells alone (left) or together with CAF cells (right). Mice were treated with vehicle, JQ1 (100 mg/ml, intraperitoneal, daily) when tumors reach 100 mm^3^. The same number of mice were used between treatments in each experiment: PDC1(*n* = 6), PDC1/CAF (*n* = 7), HT-29 (*n* = 10), and HT-29/CAF (*n* = 10). **c** Cell viability of PDC1-luc cells (left, *n* = 3), HT-29-luc cells (middle, *n* = 3), and SW480-luc cells (right, *n* = 3), which were cocultured with CAFs for 3 days, followed by treatment with indicated inhibitors for an additional 3 days. **d** Cell viability of PDC1-luc cells (left, *n* = 3) and SW480-luc cells (right, *n* = 3), which were cocultured with CAFs, followed by treatment with indicated inhibitors. **e** Immuno-deficient NOD/SCID or nude mice were engrafted with PDC1 tumor cells together with CAF cells. Mice were treated with regimen 1: vehicle, JQ1 (100 mg/ml), pacritinib (100 mg/kg), or combination or regimen 2: vehicle, JQ1 (100 mg/ml), Tocilizumab (50 mg/kg)/reparixin (150 mg/kg), or combination when tumors reach 100 mm^3^. (*n* indicates the mice number used in each group). **f** Left panel: IHC analysis of pJAK2 and pY97/98-BRD4 in representative HT-29 xenografts from **e** (Scale bars, 20 μm). Right panel: quantification of pJAK2 and pY97/98-BRD4 in left panel (*n* = 6 independent xenografts samples from each group). In **a**, **c**, and **d**, data are presented as the mean ± SEM (error bars) of three independent experiments. The *p* values were determined using two-tailed Student’s *t* tests. ns indicates non-significant.

BET inhibitors (BETi) targeting BET family of BD-containing proteins, including BRD4, have been considered as promising anti-cancer agents, but solid tumors, including CRC, are commonly resistant to BETi^29,30^. Previous studies have shown an elevated BRD4 level confers resistance to BETi^8–11^. Given that CAF induces BRD4, we next sought to determine whether CAF causes BETi resistance. To this end, we used three different small molecule BET inhibitors JQ1, MZ1, and dBET1, which are potent inhibitors of BRD4^13,14,17^. Although all the three BETi can effectively inhibit the CRC proliferation, coculture with CAFs largely diminished their effects (Fig. 5a). In vivo xenograft tumor engrafted with CRC alone showed an inhibitory response to JQ1, but this effect was diminished when CRC was co-engrafted with CAFs (Fig. 5b). These findings support the role of CAFs in promoting CRC resistance to BETi. Consistent with this hypothesis, resistance to BETis in CRC/CAF cocultures were sufficiently reversed by co-treatment with pacritinib (Fig. 5c). Similarly, blockade of IL6/8 receptors with Tocilizumab and Reparixin also abolished the resistance to BETi (Fig. 5d).

Moreover, in several xenograft tumor mouse models co-engrafted with CRC and CAF, we see JQ1 in combination with pacritinib or Tocilizumab/Reparixin resulted in remarkable suppression of the tumor growth, though the JQ1, pacritinib or Tocilizumab/Reparixin alone only produced a modest effect (Fig. 5e). The treatment showed modest effect on CAFs and the production of IL6 and IL8 (Fig. S7a, b). Consistent with the tumor suppression, the combination treatment resulted in much-reduced pJAK2 and pBRD4 in the xenograft tumors as compared with the single-agent treatment (Fig. 5f). Together, these findings demonstrate that blockade of IL6/8-JAK2 cascade could be a potential strategy to overcome BETi resistance caused by the tumor microenvironment.

### BRD4 phosphorylation at Y97/98 results in reduced binding to BETi but increased binding to chromatin

**Fig. 6:**
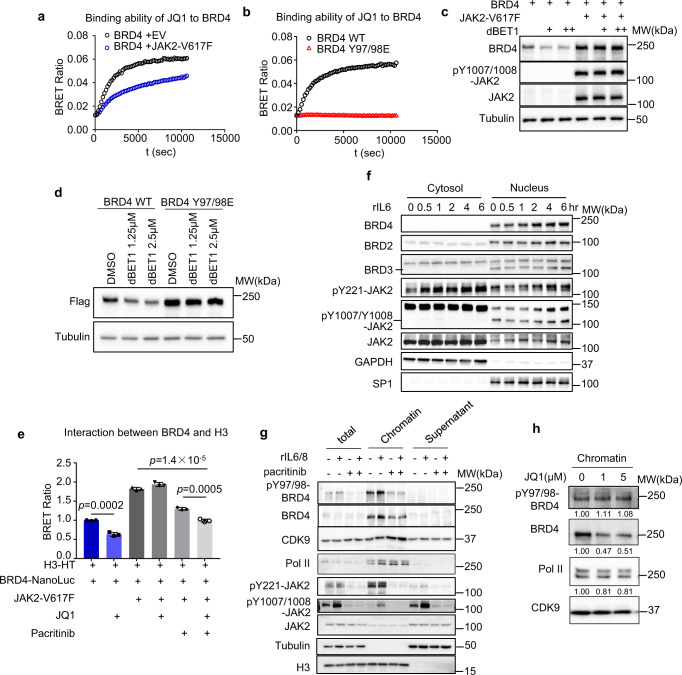
BRD4 phosphorylation at Y97/98 modulates its binding to chromatin and BET inhibitor. **a** NanoBRET report assay determining the binding ability of JQ1 to BRD4 in 293T cells transfected with BRD4 and active JAK2-V617F or empty vector. **b** NanoBRET report assay determining the binding ability of JQ1 to BRD4 in 293T cells transfected with BRD4-wild-type or BRD4-Y97/98E. **c** Representative western blot analysis (*n* = 3) of indicated proteins in 293T cells transfected with indicated constructs and treated with dBET1(1.25 μM, 2.5 μM). **d** Representative western blot analysis (*n* = 3) of indicated proteins in 293T cells transfected with flag-BRD4 or flag-BRD4-Y97/98E followed by dBET1 treatment. **e** NanoBRET™ BRD4/Histone H3.3 Interaction Assay in 293T cells determining the interaction between BRD4 and histone H3.3 under different conditions. 293T cells were transfected with BRD4-NanoLuc and histone H3.3-HaloTag with/without active JAK2-V617F as indicated, then were treated with indicated inhibitors. **f** Representative western blot analysis (*n* = 2) of indicated proteins from cytosol and nucleus of PDC1 cells treated with 20 ng/ml rIL6 for indicated time. **g** Representative western blot analysis (*n* = 3) of indicated proteins from WCL, chromatin, and supernatant of PDC1 cells treated with 50 ng/ml rIL6/8 and/or 2.5 μM pacritinib. **h** Representative western blot analysis (*n* = 2) of indicated protein from chromatin of PDC1 cells treated with 20 ng/ml rIL6 followed by JQ1 treatment. In **e**, data is presented as the mean ± SD (error bars) of three biological replicates. The *p* values were determined using two-tailed Student’s *t* tests.

Bromodomains (BD1 and BD2) of BRD4 is required for the recognition and binding of the acetylated lysine residues of histones and other cellular proteins to execute its function as a chromatin regulator. It is interesting to notice that Y97 has been previously reported to reside in the binding pocket of JQ1, which is important for JQ1 to inhibit BRD4 activity^16,17^. Our finding thus raised an intriguing possibility that JAK2-induced BRD4 phosphorylation at Y97/98 may affect JQ1 binding to BRD4, thus resulting in resistance to JQ1. To test this hypothesis, we performed a NanoBRET Reporter assay in which JQ1 binding affinity to Nanoluc-BRD4 fusion protein can be measured by a BRET luminescent tracer in a cellular system^31^. We found that 293T cells transfected with Nanoluc-BRD4 alone showed a high binding activity to JQ1, which was markedly reduced in cells that were co-transfected with JAK2-V617F (Fig. 6a). Moreover, in contrast to the Nanoluc-BRD4, Nanoluc-BRD4-Y97/Y98E mutant transfected in the cells showed a complete loss of its binding to JQ1 (Fig. 6b). Together, these data demonstrated the Y97/98 is required for JQ1 binding, whose phosphorylation by JAK2 ablated its binding to JQ1. To further consolidate this finding, we also used a BRD4 degrader, dBET1, which is a proteolysis-targeting Chimera (PROTAC) version of JQ1 that binds to BRD4 and induces BRD4 degradation^13^. As expected, though dBET1 with increasing concentrations can cause BRD4 degradation, it failed to do that in cells co-transfected with the JAK2-V617F (Fig. 6c). Likewise, BRD4 phosphorylation mimic BRD4-Y97E/Y98E also showed resistance to dBET1-induced degradation (Fig. 6d). Together, these findings using different approaches have demonstrated that JAK2-induced BRD4 phosphorylation at Y97/98 abolished its binding to BETi.

BRD4 regulates chromatin activity through binding to acetylated histone^32^. Next, we performed the NanoBRET assay to monitor the proximity of BRD4 and Histone 3.3 (H3.3) in the context of JAK2 activation. As expected, the interaction between BRD4 and H3.3 was reduced by JQ1 through competitive binding but increased by JAK2-V617F (Fig. 6e). In the presence of JAK2-V617F, the BRD4-Histone 3 interaction was no longer sensitive to JQ1, but this was reversed by pacritinib (Fig. 6e). Interestingly, cell fractionation analysis showed that rIL6 induced nuclear accumulation of pJAK2 (Fig. 6f); It also caused the enrichment of pJAK2, along with BRD4 and pBRD4 in the chromatin fraction, which can be diminished by pacritinib (Fig. 6g). Moreover, although JQ1 was able to displace the BRD4 protein from the chromatin as expected, it could not replace the phosphorylated BRD4 from the chromatin (Fig. 6h). These data demonstrated that JAK2-induced phosphorylation of BRD4 results in a favored binding to chromatin while reducing binding to JQ1. These observations have provided a plausible explanation for the BETi resistance in tumors enriched with an inflammatory tumor microenvironment.

### pBRD4 interacts with STAT3 to confer increased oncogenic enhancer activity

**Fig. 7:**
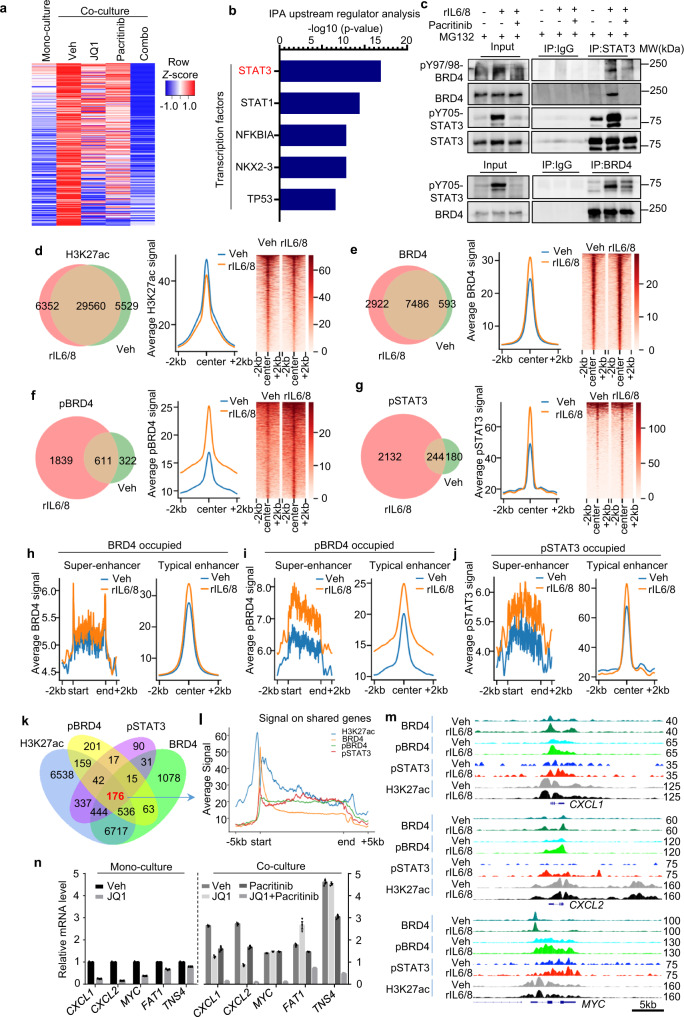
BRD4 phosphorylation promotes interaction with pSTAT3 to induce chromatin reprogramming with increased oncogenic enhancer activity. **a** Heatmap showing 371 genes which were upregulated (more than 2 folds, *p* < 0.05) by coculture with CAF in PDC1, but suppressed (more than 2 folds, *p* < 0.05) by combination of pacritinib and JQ1. **b** IPA upstream regulator analysis of 371 genes in **a** showing top5 transcription factors. The top upstream regulator was highlighted in red. **c** Representative western blot analysis (*n* = 2) of WCL and immunoprecipitates by anti-BRD4 or anti-STAT3 antibodies from PDC1 cells treated with 50 ng/ml rIL6/8 and/or 2.5 μM pacritinib together with 20 μM MG132 as indicated. **d**–**g** Positional overlap (left panel) of chromatin binding, average intensity curves (Middle panel) and heatmaps (right panel) of ChIP-seq reads (RPKM; reads per kilobase of transcript per million mapped reads) for H3K27ac (**d**), BRD4 (**e**), pBRD4 (**f**), and pSTAT3 (**g**) before and after rIL6/8 treatment based on ChIP-seq in PDC1 cells. **h**–**j** Average intensity curves of ChIP-seq reads for BRD4 (**h**), pBRD4 (**i**), and pSTAT3 (**j**) at super-enhancer (left panel) and typical enhancer (right panel) before and after rIL6/8 treatment based on ChIP-seq in PDC1 cells. **k** Venn diagram showing the overlap genes occupied by H3K27ac, BRD4, pBRD4, and pSTAT3 on their chromatin under rIL6/8 treatment in PDC1 cells. 176 common genes were identified. **l** Average intensity curves of ChIP-seq reads from 176 common genes from **k** for H3K27ac, BRD4, pBRD4, and pSTAT3. **m** Genome browser view of normalized ChIP-seq signals of BRD4, pBRD4, pSTAT3, and H3K27ac at the CXCL1, CXCL2, and MYC locus in vehicle and rIL6/8 treated PDC1 cells. **n** qRT-PCR analysis (*n* = 2) of indicated genes in the PDC1 cells which were treated with indicated chemicals under mono-culture or coculture conditions. Data is presented as the mean ± SEM (error bars) of technical triplicates.

To identify the key transcriptional program induced by CAF-activated JAK2-BRD4 signaling, we performed a transcriptome analysis in PDC cocultured with or without CAF and treated with pacritinib, JQ1 or both. We identified 371 genes whose expression were upregulated by CAF but reduced by either JQ1, pacritinb or both (using 2-fold cutoff, *p* ≤ 0.05) (Fig. 7a). Consistent with the combination effect of JQ1 and pacritinib in CRC growth, we found that the majority of this gene set showed synergistic downregulation upon the combination treatment, indicating that this gene set is regulated cooperatively by JAK2 and BRD4. Not to our surprise, ingenuity analysis of upstream regulators reveals that STAT3 is the top upstream regulator of this gene set (Fig. 7b). Given that STAT3 has been previously shown to associate with other epigenetic regulators to modulate chromatin activity^33^, we asked whether STAT3 acts as a transcriptional effector of JAK2 to coordinate with BRD4 to regulate chromatin activity. For this reason, we performed reciprocal co-immunoprecipitation and uncovered a steady interaction between pSTAT3 and pBRD4 in PDC cells treated with rIL6/8, which can be disrupted by pacritinib (Fig. 7c). These data suggest that STAT3 activation by IL6/8 leads to association with pBRD4 to regulate the chromatin activity coordinately.

BRD4 is known to bind to gene enhancers and super-enhancers to regulate gene expression^34,35^. To understand the association between BRD4 and pSTAT3 at the chromatin level, we performed chromatin immunoprecipitation coupled with deep sequencing (ChIP-seq) for the total BRD4, pBRD4, pSTAT3, and the active enhancer marker H3K27ac in PDC cells treated with or without rIL6/8. Although rIL6/8 treatment did not induce ChIP-seq signal of total H3K27ac mark (Fig. 7d), it significantly increased the pBRD4 and pSTAT3 binding to chromatin. In contrast, there was only a modest change of the total BRD4 binding to chromatin (Fig. 7e–g). This observation indicates that in response to IL6 and IL8 signals, pBRD4 and pSTAT3 regulate chromatin activity distinctly from BRD4.

We further characterized the rIL6/8 response on typical enhancers and super-enhancers^36^ (Fig. S8a). Similar to the overall H3K27ac signals (Fig. 7d), rIL6/8 treatment did not seem to induce apparent changes in the total super-enhancers and typical enhancers (Fig. S8a, b). However, rIL6/8 treatment induced much-increased super-enhancers or typical enhancer signals bound by pBRD4, pSTAT3, but to a lesser degree BRD4 (Fig. 7h–j). Further analysis identified 176 sites that are commonly regulated by BRD4, pBRD4, pSTAT3, and H3K27ac in response to rIL6/8 treatment, suggesting a set of enhancers concurrently regulated by BRD4, pBRD4, and pSTAT3 (Fig. 7k). Signal distribution analysis of the associate genes showed that although BRD4 seemed to bind mainly to the promoter region of these genes, pBRD4 and pSTAT3 showed widespread binding to enhancers across the gene bodies (Figs. 7l and S8c). Interestingly, several well-known oncogenes linked to aggressive and metastatic cancer phenotype, including *CXCL1*, *CXCL2*, *MYC*, and *BMP4*, were included in this gene get, suggesting that BRD4 and STAT3 can coordinately regulate the oncogenic enhancer activity to promote tumor progression. Of note, these oncogenes were occupied by extended enhancer clusters showing H3K27ac, pBRD4, and pSTAT3 peaks across the gene bodies, while BRD4 is more found in the promoter regions (Fig. 7m).

Thus, these findings suggest that paracrine inflammatory signals promote pBRD4 and pSTAT3 interaction to augment enhancer activity towards more robust oncogenic transcription. Consistent with this notion, RT-PCR validation of several of these oncogenes showed that CAFs induced their expression, which required a combination of JQ1 and pacritinib to abolish their expression (Fig. 7n) effectively. ChIP-qPCR analysis showed that the binding of pBRD4 on the promoters of *CXCL1*, *CXCL2*, *MYC* was more resistant to JQ1 treatment comparing to that of BRD4, and the combination of JQ1 and pacritinib largely diminished the binding (Fig. S8d). Taken together, these data indicate tumor microenvironment provides a mechanism to promote the pBRD4 and pSTAT3 association to increase enhancer activity towards oncogenic transcription and a co-targeting strategy is necessary to abolish or revert this process.

## Discussion

We demonstrate a previously unrecognized tumor microenvironment mechanism by which paracrine IL6/IL8-JAK2 signaling induces BRD4 activation in CRC, leading to chromatin remodeling and resistance to BETi treatment. Previous studies have documented the role of stromal infiltration, as well as CAF-secreted IL6/IL8 and associated JAK2-STAT3 signaling in CRC relapse and poor prognosis^20,37–41^. Our finding highlights important crosstalk between the inflammatory signaling in the tumor microenvironment and the chromatin regulatory machinery in cancer cells, which provides insights into the understanding of the CRC progression.

Several BETis are under clinical development, but the clinical trial results have been less encouraging in most solid tumors. Several recent studies have reported mechanisms of BETi resistance, but these studies have focused on intrinsic cancer mechanisms. Among them, multiple serine phosphorylation of BRD4 modulated by CK2, CDK9, or PP2A phosphatase at nearby the BD2 domain has been shown to increase BRD4 chromatin binding capacity or promotes JQ1 resistance by facilitating bromodomain-independent chromatin recruitment^7,42,43^. In addition, BRD4 stabilization due to genetic loss of function of E3 ubiquitin ligase SPOP has been associated with resistance to BETi in prostate cancer^9–11^. Our study, however, provides a different mechanism by which the tumor microenvironment induces BRD4 stabilization through tyrosine phosphorylation at BD1 domain at Y97/98.

Interestingly, Y97 has been previously shown to be within the binding pocket of JQ1^16^. Our finding that Y97/98-phosphorylated BRD4 has increased activity binding to chromatin but reduced binding to JQ1 has provided at least partially a mechanistic explanation for the lack of response to BETi in solid tumors. Considering that BETi resistance is common in solid tumors that are featured with an inflammatory TME, we envision that this mechanism is widely applicable to other cancer as well.

Mechanistically, through comprehensive biochemical screening and tumor samples profiling, we identified UCHL3 as a crucial deubiquitinase that binds explicitly to JAK2-phosphorylated BRD4 to maintain its stabilization. Significantly, UCHL3 is overexpressed in CRC, supporting it being a relevant DUB to maintain a high level of BRD4 protein expression in CRC. Of note, new generation of BETi, BET-PROTACs have been designed to degrade BRD4 avoiding resistance induced by BRD4 stabilization through hijacking the ubiquitin E3 ligase. However, the resistance to BET-PROTACs has also emerged. We thus propose that UCHL3 may serve as an additional target to block BRD4 signaling or re-sensitize CRC response to BETi, including BET-PROTACs, which warrant a further investigation. Interestingly, a recent study shows that a proficient expression of deubiquitinase DUB3 in prostate cancer can promote BRD4 stabilization and resistance to BETi^8^. Therefore, the deubiquitinases involved in the regulation of BRD4 stability appear to be cancer-dependent.

An important finding in this study is the demonstration that IL6/8-JAK2-induced pBRD4 physically associates with pSTAT3. Consistent with this biochemical finding, we show that pBRD4 and pSTAT3 co-localize on the enhancers across the entire gene bodies of some of the key oncogenes such as *MYC*, *CXCL1*, and *CXCL2*, which exhibit increased enhancer-binding activity in response to IL6 and IL8. By contrast, we found that BRD4 is mainly found on the promoters of these genes and is less responsive to IL6 and IL8 treatment. These findings suggest that phosphorylated BRD4 displays a gain of distinct enhancer activity through association with pSTAT3. This feature of TME-induced chromatin remodeling establishes pBRD4 and JAK2-STAT3 signaling to convergence on a set of crucial oncogenes whose hyperactive transcription state is believed to be necessary for disease progression. Indeed, we show that either increased JAK2 or pBRD4 in CRC is associated with metastasis and poor survival of CRC patients.

The corroborative regulation of some of the important oncogenes known to be important in cancer progression by both pBRD4 and pSTAT3 suggests that a co-targeting strategy is necessary to inhibit the oncogenic transcription. Indeed, we show that combined inhibition of BRD4 and IL6/8-JAK2 signaling is required to inhibit oncogene expression more completely, reversed the BETi resistance in vitro, and suppress the CRC xenograft tumor growth empowered by CAF in vivo. In our pre-clinical models, the combination of JQ1 with pacritinib or IL6/IL8 targeting agents did not have observed adverse effects, with no obvious toxicity in mice. Indeed, pacritinib is currently in late-stage clinical development, and tocilizumab has already been in clinical use. Therefore, the pharmacologic blockage of JAK2-induced BRD4 activity through the aforementioned combination approach has the potential of being translatable into a clinical setting. In this clinical setting, assessing the expression of pJAK2, pBRD4, and UCHL3 in CRC may be useful to guide the patient selection for an appropriate treatment strategy. Taken together, we have demonstrated a tumor microenvironment-mediated mechanism that induces chromatin reprogramming and contributes to resistance to BETi, illustrating the potential of developing a therapeutic strategy to target the resistance mechanism.

## Methods

### Generation of patient-derived cancer cells and cancer-associated fibroblasts models

The primary human biological samples were obtained from individuals treated at the Sixth Affiliated Hospital, Sun Yat-sen University (Guangzhou, Guangdong, China)^28^. All the studies with these samples were under informed consent and approved for research purposes by the Ethics Committee of the Sixth Affiliated Hospital, Sun Yat-sen University. For expansion of tumor spheres, the resected primary human colorectal tumors were washed with PBS-supplemented Antibiotic-Antimycotic (Invitrogen, Thermo Fisher Scientific, Waltham, MA), minced with a sterile blade, and were digested in DMEM/F12 medium supplemented with 1 mg ml^−1^ collagenase/dispase (Roche, Indianapolis, IN) at 37 °C for 1 h. The cell suspensions were then filtered through 70 and 40 μm cell strainers (BD Falcon, San Jose, CA), followed by depletion of erythrocytes using 1 × RBC Lysis Buffer (eBioscience # 00-4333-57). The cells were then washed with PBS once and maintained in six-well ultra-low attachment plate (Corning #CLS3471) in CSC medium [serum-free DMEM/Ham’s F12 (Nacalai Tesque #08460-95), supplemented with N2 (Invitrogen #17502-048), B27 (Invitrogen #12587-010), 20 ng ml^−1^ basic-fibroblast growth factor (bFGF) (BD Biosciences), 20 ng ml^−1^ epidermal growth factor (EGF), 4 µg ml^−1^ heparin (Stemcell Technologies #07980), and 0.5 µg ml^−1^ hydrocortisone (Sigma-Aldrich #H0888)]. The monolayer patient-derived cancer cells (PDC) were generated from the tumor spheres which were dissociated into single cells, then placed in normal cell culture dishes in DMEM medium (Gibco) supplemented with 10%FBS, NEAA (Gibco, #11140050), and insulin (Sigma, #I2643).

For isolation and establishment of primary fibroblasts, primary colorectal tumors tissues were minced with a sterile blade and incubated with 1 mM EDTA (Sigma) at 37 °C for 30 min with repetitive shaking to remove epithelial cells. The samples were further digested in 1 mg ml^−1^ collagenase/dispase (Roche) in DMEM/F12 medium at 37 °C for 1 h with shaking. The cells were washed with PBS once and plated at high density with DMEM supplemented with 10% fetal bovine serum (FBS). After 4–6 passages, the fibroblasts were harvested and subjected to quantitative RT-PCR analysis of universal fibroblasts markers (*VIM* and *TWIST*) and cancer-associated fibroblast (CAF) marker (*α-SMA*)^28^. The immunocytochemistry (ICC) analysis further confirmed the expression level of known CAF markers, including α-SMA, vimentin, and fibronectin, were higher in CAFs compared to normal fibroblasts (NFs). All media were supplemented with 5000 U ml^−1^ penicillin/streptomycin (Invitrogen). All cells were maintained at 37 °C with 5% CO_2_.

### Cell lines and reagents

All other cell lines were obtained from American Type Culture Collection (ATCC). All cell lines were tested negative for mycoplasma contamination. HT-29 (ATCC, Cat#HTB-38, RRID: CVCL_0320) and SW480 (ATCC, Cat#CCL-228, RRID:CVCL_0546) colon cancer cell lines, MCF7 (ATCC, Cat# HTB-22, RRID: CVCL_0031) breast cancer cell lines, H1299 (ATCC, Cat#CRL-5803, RRID:CVCL_0060) and A549 (ATCC, Cat#CCL-185, RRID:CVCL_0023) lung cancer cell lines, DU145 (ATCC, Cat#HTB-81, RRID:CVCL_0105) and LNCaP (ATCC, Cat #CRL-1740,RRID: CVCL_1379) prostate cancer cell lines, and HEK293T (ATCC, Cat #CRL-11268, RRID: CVCL_1926) cell line were grown in DMEM supplemented with 10% FBS. All media were supplemented with 5000 U/ml penicillin–streptomycin. All cell lines were maintained at 37 °C in a humidified atmosphere with 5% CO_2_. Cell culture reagents and other chemicals were purchased from Thermo Fisher Scientific (Gibco & Hyclone), Corning, and Sigma-Aldrich.

The following recombinant proteins were used in cell treatments: Recombinant human IL-6 (Sigma-Aldrich, Cat#I1395), Recombinant human IL-8 (PeproTech, Cat#200-08), human TNFα (PeproTech, Cat#300-01 A), human IL-1β (PeproTech, Cat#200-01B), Recombinant human TGF-β1 (Miltenyi Biotec, Cat#130-095-067), IL17α (PeproTech, Cat#200-17), GM-CSF (PeproTech, Cat#300-03), CXCL12 (PeproTech, Cat#300-28 A), CCL2 (PeproTech, Cat#250-10), CCL5 (PeproTech, Cat#300-06), CCL7 (PeproTech, Cat#250-08), S100A7 (ProSpec, Cat#pro-149), and S100A8 (ProSpec, Cat#pro-800). And the following compounds were used in in vitro assay according to the manufacturer’s instructions: MG132 (Selleck Chemicals, Cat#S2619), Cycloheximide (Sigma-Aldrich, Cat#C-7698), JQ1 (MedChemExpress, Cat#HY-13030), MZ1 (MedChemExpress, Cat#HY-107425), dBET1 (MedChemExpress, Cat#HY-101838), and Pacritinib (MedChemExpress, Cat#HY-16379).

### Quantitative coculture assays

For the cell viability assay using direct cell–cell contact coculture, the colon PDC cells, HT-29 or SW480 stably expressing luciferase (PDC-luc, 1 × 10^3^ cells), (HT-29-luc, 1 × 10^3^), or (SW480-luc, 1 × 10^3^) cells were overlaid directly on CAFs or NFs. Briefly, CAFs or NFs (3 × 10^3^ cells) were seeded in the collagen-coated (type I collagen, Sigma-Aldrich; catalog number: C8919) white flat bottom 96-well plate for 3 days as a monolayer. Next, the PDC-luc, HT-29-luc or SW480-luc were directly added onto fibroblasts using specified N2 medium [serum-free DMEM/Ham’s F12 (Nacalai Tesque, Inc.) and 1 × N-2 supplement (Gibco)] and DMEM supplemented with 10% FBS, respectively, in (5% CO_2_) incubator for 3 days. The cocultured cells were then treated with DMSO solvent control or various concentrations of single and/or combined targeted inhibitors for additional 3 or 5 days. The cell viability of PDC-luc, HT-29-luc or SW480-luc cells were measured by luciferase activity when adding 1 × luciferase substrate, luciferin (Promega, Madison, WI), for which chemiluminescent signal was detected with a microplate reader.

For the separate coculture, the PDC cells (1 × 10^5^ cells) were seeded in the flat bottom 24-well plate (Corning Falcon, catalog number: 353504), while CAFs or NFs (2 × 10^4^ cells) were plated in the collagen-coated 24-well chamber insert (8 µm, Corning Falcon, catalog number: 353097). After 1-day incubation, the insert was put into 24-well plate and medium was replaced with DMEM supplemented with 10% FBS. In some experiments, the cocultured cells were treated with dBET1 (0.5 μM) for 18 h with Pacritinib (2.5 μM) treatment at the final 6 h. In some experiments, the cocultured cells were maintained for 48 h and were treated with Pacritinib (1 μM) at the final 24 h.

### Conditioned media collection and ELISA assay

For the collection of conditional medium (CM), CAFs or NFs (1.25 × 10^5^ cells) were seeded in the collagen-coated flat bottom six-well plate and maintained in DMEM medium (Gibco) containing 1% or 10% FBS (Gibco) for 48 h. The medium was harvested and centrifuged at 800 × *g* for 5 min. The clear supernatant was then collected as CM.

For detection of the amount of secreted human IL-6, IL-8 in CAF-CM, or NF-CM, the enzyme-linked immunosorbent assay (ELISA) assay was performed using ELISA assay kit purchased from Boster Biological Technology: IL6 ELISA kit (Boster, Cat#EK0410), IL8 ELISA kit (Boster, Cat#EK0413). Dilution (1:10) was performed on the CM before quantifying the amount of cytokines according to the manufacturer’s protocol.

### Prediction of phosphorylation of BRD4 by JAK2

For prediction of the JAK2-mediated phosphorylation site of BRD4, the prediction algorithm of the Group-based Prediction System (GPS 3.0) was used (http://gps.biocuckoo.org)^23^. The result showed that the Y97, Y98, Y390, and Y599 tyrosine residues in BRD4 were potentially phosphorylatable by JAK2.

### Generation of phosphor-Y97/Y98-BRD4 antibody

For the generation of phosphor-Y97/Y98-BRD4 antibody, the dual-phosphor-(Y97/Y98) BRD4 peptide (CNLPD-pY-pY-KIIKT) was synthesized and injected into the rabbit once a week according to the manufacturer’s standard procedures (iDNA Biotechnology Pte. Ltd., Singapore). Rabbits were killed to collect serum-containing antibodies after 3 months post-injection. Affinity purification was performed according to the manufacturer’s standard procedures (Singapore Advanced Biologics Pte. Ltd., Singapore). To validate the specificity using western blots analysis, the dual-phosphor-(Y97/Y98) BRD4 antibody (dilution 1:500) was incubated with the membrane containing BRD4 non-phosphor peptide (CNLPDYYKIIKT) or phosphor peptide (CNLPD-pY-pY-KIIKT) overnight at 4 °C, followed by secondary antibody, anti-rabbit IgG conjugated with horseradish peroxidase.

### In vitro kinase assay

For validation of JAK2-mediated phosphorylation of BRD4, we performed in vitro kinase assay using the pulled-down constitutively active myc-tag-JAK2-V617F or kinase-dead JAK2-K882R from 293T cells and two recombinant human BRD4 peptides (a.a. 44-168 or a.a. 333-460) (Active Motif #31380 and #31446). The 250 ng of each BRD4 peptides were incubated with agarose-bound myc-tag-JAK2 in the kinase assay buffer (10 mM HEPES, 50 mM NaCl, 5 mM MgCl_2_, 5 mM MnCl_2_, 0.1 mM Na_3_VO_4_, and 1 mM NaF) supplemented with 1 mM DTT and 2.5 mM ATP (New England Biolabs #P0756) at 30 °C for 30 min. The reaction was stopped by adding the 3× SDS loading buffer and heated to 100 °C for 5 min. Samples were then separated by 4–15% SDS-page, followed by western blotting using a pan-Phospho-Tyrosine antibody (Cell Signaling #9411) or dual phospho-Y97/Y98-BRD4 antibody (iDNA Biotechnology).

For validation of Y97/Y98 of BRD4 is phosphorylated by JAK2, the in vitro kinase assay was performed using the pulled-down flag-tag-BRD4-WT or BRD4-Y97A/Y98A from 293T cells and the recombinant active JAK2 protein (antibodies-online.com #ABIN5508975). In total, 250 ng active JAK2 were incubated with agarose-bound Flag-tag-BRD4 in kinase assay buffer (10 mM HEPES, 50 mM NaCl, 5 mM MgCl_2_, 5 mM MnCl_2_, 0.1 mM Na_3_VO_4_, and 1 mM NaF) supplemented with 1 mM DTT and 2.5 mM ATP (New England Biolabs) at 30 °C for 30 min, followed by western blotting as described above.

### LC-MS/MS analysis for phosphorylated site of BRD4

To investigate which residues of BRD4 could be phosphorylated by JAK2, we performed in vitro kinase assay using active JAK2 (antibodies-online.com #ABIN5508975) and recombinant human BRD4 peptides (a.a. 44-168, BD1) as described above. The reaction was stopped by adding the 3 × SDS loading buffer and heated to 100 °C for 5 min. Samples were then separated by 4-15% SDS-page, followed by staining with Coomassie Blue (ThermoFisher, #24615). Then the BD1 peptides were identified by the LC-MS/MS analysis, which was performed by PTM BIO (Hangzhou, China).

### Quantitative RT-PCR

Total RNA was isolated by using TRIzol (Invitrogen) and purified with the RNeasy Mini Kit (Qiagen, Valencia, CA). Reverse-transcription and quantitative PCR assays were performed using High-Capacity cDNA Reverse Transcription Kit and KAPA SYBR Fast qPCR kit (KAPA Biosystems, Wilmington, MA). For quantification of mRNA levels, *ACTB* or *GAPDH* level was used as an internal control. All reactions were analyzed in an Applied Biosystems PRISM 7500 Fast Real-Time PCR system in 96-well plate format. Primer sequences for specific genes are shown in Supplementary Table 1.

### Western blot

Cells were lysed using RIPA buffer, in the presence of protease inhibitor set (Roche #11836170001) and phosphatase inhibitor cocktail set (Roche #4906845001). Protein concentration was measured with Bio-Rad Protein Assay Kit (Bio-Rad #5000006) following manufacturer’s instructions. Two volumes of proteins were mixed with one volume of 3 × Blue Loading Buffer (Cell Signaling Technology #7722) and boiled at 95 °C for 5 min. Proteins were run on pre-cast gels (Bio-Rad #4568096 or #4568086) or home-made SDS-Page gels. Precision Plus Protein™ Dual Color Standards was used (Bio-Rad #1610374). Gels were run using 1× running buffer (Bio-Rad #1610772). Gels were transferred using 1 × transfer buffer (Bio-Rad #1610771). PVDF membrane paper (Immobilon #IPVH00010) was activated in 100% methanol and was used for transfer. The membrane was then blocked with 5% non-fat milk or 5% BSA for 1 h prior to addition of primary antibody. Membrane was washed with 1 × TBST. The following primary antibodies were used according to the manufacturer’s instructions: BRD4 (Cell Signaling, Cat#13440, RRID:AB_2687578), pY97/98-BRD4 (homemade, iDNA), JAK2 (Cell Signaling, Cat#3230, RRID:AB_2128522), phospho-JAK2(Tyr221) (Cell Signaling, Cat#3774, RRID:AB_390750), Myc-tag (Cell Signaling, Cat#2278, RRID:AB_490778), HA-tag (Cell Signaling, Cat# 5017, RRID:AB_10693385), STAT3 (Cell Signaling, Cat#9139, RRID:AB_331757), pan-phospho-Tyrosine (Cell Signaling, Cat#9411, RRID:AB_331228), phospho-JAK2 (Tyr1007/1008) (Abcam, Cat#ab32101, RRID:AB_775808), phospho-STAT3 (Tyr705) (Abcam, Cat#ab76315, RRID:AB_1658549), BRD2 (ProteinTech, Cat#22236-1-AP, RRID:AB_2722524), BRD3 (ProteinTech, Cat#11859-1-AP, RRID:AB_2065902), SPOP (ProteinTech, Cat#16750-1-AP, RRID:AB_2756394), β-actin (Sigma-Aldrich, Cat#A5441, RRID:AB_476744), Flag-tag (Sigma-Aldrich, Cat#F1804, RRID:AB_262044), G9A (Upstate, Cat#07-551, RRID:AB_310709), SMYD3 (Abcam, Cat#ab16027, RRID:AB_777995), DNMT1 (Alexis Biochemicals, Cat#ALX-804-369-C100, RRID:AB_2051311), DNMT3b (Pierce, Cat#PA1-884, RRID:AB_2277463), HDAC1 (Upstate, Cat#05-614), HDAC2 (Upstate, Cat#05-814, RRID:AB_310022), JMJD3 (Abcam, Cat#ab38113, RRID:AB_943898), EZH2 (Cell Signaling, Cat#5246, RRID:AB_10694683), H3K9Ac (Upstate, Cat#07-352, RRID:AB_310544), H3K14Ac (Upstate, Cat#07-353, RRID:AB_310545), H3K27Ac (Abcam, Cat#ab4729, RRID:AB_2118291), H3K27Me3 (Abcam, Cat#ab6002, RRID:AB_305237), H3K4Me3 (Millipore, Cat#07-473, RRID:AB_1977252), H3K9Me3 (Abcam, Cat#ab8898, RRID:AB_306848), H3K36Me3 (Abcam, Cat#ab9050, RRID:AB_306966), H3 (Cell Signaling, Cat#3638, RRID:AB_1642229), His-tag (Abcam, Cat#ab18184, RRID:AB_444306), UCHL3 (Abcam, Cat#ab241490), Pol II (Sigma-Aldrich, Cat#04-1569, RRID:AB_10615427), CDK9 (Sigma-Aldrich, Cat#MABF770), SP1 (Millipore, Cat#07-645, RRID:AB_310773), and β-Tubulin (Sigma-Aldrich, Cat#T4026, RRID:AB_477577). Horseradish peroxidase-linked secondary antibodies: anti-mouse IgG (NA931-1ML) and anti-Rabbit IgG (NA934-1ML) were acquired from GE Healthcare. All primary antibodies for western blot were used in a dilution of 1:1000, except anti-β-actin used in 1:5000, and the secondary antibodies were used in 1:10,000 dilution. The uncropped scans for all western blot are provided in the Source Data file.

### Co-immunoprecipitation

Co-immunoprecipitation (Co-IP) assays were performed as described previously^44,45^ with a modified protocol using NETN buffer (25 mM Tris-HCl, 150 mM NaCl, 1 mM EDTA, 10% glycerol, 0.5% NP40) containing Protease Inhibitor cocktail (Roche #11836170001) and phosphatase inhibitor cocktail (Roche #4906845001) to extract whole-cell protein, or a cell fractionation protocol for cytoplasmic and nuclear fractionation using NE-PER™ Nuclear and Cytoplasmic Extraction Reagents (ThermoFisher Scientific #78833). Cell lysates were incubated with the indicated antibodies at 4 °C overnight. The protein complex was captured using protein A-agarose or protein G-agarose beads (Roche, Indianapolis, IN, USA) at 4 °C for 4 h, and agarose beads were collected by centrifuge and washed three times with washing buffer. The precipitated proteins were mixed with 1× Blue Loading Buffer along with 3 mM dithiothreitol (Cell Signaling Technology #7722) and subjected to western blot analysis. The following primary antibodies were used for Co-IP: BRD4 (Cell Signaling, Cat#13440, RRID: AB_2687578), HA-tag (Cell Signaling, Cat# 5017, RRID: AB_10693385), Flag-tag (Sigma-Aldrich, Cat#F1804, RRID:AB_262044), and STAT3 (Cell Signaling, Cat#9139, RRID:AB_331757).

### BET inhibitors and BRD4 binding assay (NanoBRET Reporter assay)

The NanoBRET Reporter assay was performed following manufacturer’s instructions (Promega #N2130). Briefly, 4 × 10^5^ 293T cells were seeded in 12-well cell culture plate. Next day, cells were transfected with transfection carrier DNA and vectors sets: NanoLuc®-BRD4 Fusion Vector together with JAK2^V617F^ or empty vector (Fig. 6a) and NanoLuc®-BRD4 Fusion Vector or NanoLuc®-BRD4-Y97/98E Fusion Vector (Fig. 6b) using lipofectamine 2000 transfection reagent. In all, 36 h after transfection, cells were harvested with trypsin and pellet cells were collected at 200 × *g* for 5 min. Cells were resuspended to density of 2 × 10^5^ cells/ml using prewarmed Assay Medium (Opti-MEM® I Reduced Serum Medium, no phenol red, and no serum, Life Technologies #11058-021). Dispense 95 µl per well of cell suspension in triplicate into white, nonbinding surface (NBS) 96-well plates (Corning # 3600). In all, 5 µl of Complete 20× NanoBRET™ Tracer Reagent per well was added to cells. After mix on an orbital shaker for 15 s at 700 rpm, 50 µl of 3× Complete Substrate plus Inhibitor Solution was then added to each well of the 96-well plate. The cells were incubated for 2–3 min at room temperature. Then the measurement was performed using the GloMax® Discover System to measure donor emission wavelength (450 nm) and acceptor emission wavelength (610 nm). For kinetic NanoBRET measurements, 60 measurements were carried out with 3-min intervals between each measurement. BRET ratios were calculated by dividing the acceptor emission by the donor luminescence.

For BRD4 and Histone3.3 binding assay, the 293t cells were transfected with Histone H3.3-HaloTag® Fusion Vector, NanoLuc®-BRD4 Fusion Vector, and JAK2^V617F^ as indicated in Fig. 6e. In all, 36 h after transfection, cells were harvested and pellet cells were collected at 200 × *g* for 5 min. Cells were resuspended to 2 × 10^5^ cells/ml using prewarmed Assay Medium, then vehicle, 2 μM JQ1 and/or 2.5 μM pacritinib were added to the cells which were incubated in the incubator with 5% CO_2_ at 37 °C for 6–8 h. Then the measurement was performed using the GloMax® Discover System to measure donor emission wavelength (450 nm) and acceptor emission wavelength (610 nm).

### Chromatin fractionation

Chromatin fractionation assay was performed according to the previous report^46^. Briefly, cells were washed in ice-cold PBS, then scraped and resuspended in chromatin extraction buffer (20 mM Tris-HCl, 100 mM NaCl, 5 mM MgCl_2_, 10% glycerol, 0.2% IGEPAL CA-630, 2 mM NaF, 2 mM Na_3_VO_4_, Protease Inhibitor Cocktail, Phosphatase Inhibitor Cocktail, pH 7.5). Cells were lysed for a total of 1 hr (cell lysate was vortexed for 5 s every 10 min). The insoluble fraction was precipitated by centrifugation (16,000 × *g*, 5 min, 4 °C) and washed three times in chromatin extraction buffer in which it was subsequently resuspended. Total cell fraction and supernatant were sampled before and after the first precipitation respectively. All fractions were supplemented with SDS (0.1% sodium dodecyl sulfate), digested with benzonase (Merck Millipore, 30 min, 4 °C) and re-dissolved by sonication in a Branson Digital Sonifier.

### Retroviral transduction and plasmids/siRNAs transfection

For the generation of patient-derived cancer cells (PDC) stably expressing luciferase (PDC-luc), parental PDC cells were infected with lentivirus packaged with pLenti-V5-luc plasmid for 48 h, followed by blasticidin (10 µg ml^−1^) selection. For the generation of doxycycline-dependent inducible *BRD4* knocked-down PDC-luc cells, three different short hairpin RNAs (shRNAs) sequences were inserted into pLV-H1TetO-RFP-Puro of the lentivirus system, and were used to knockdown *BRD4* genes. The sh*LacZ* was used as control vector. The lentivirus-infected cells were then maintained in CSC medium supplemented with puromycin (2 µg ml^−1^) for selection.

For the generation of the wild-type human BRD4 (BRD4-WT) or mutant BRD4 overexpressing plasmid, the full-length WT *BRD4* DNA were subcloned from pcDNA5-Flag-BRD4-WT plasmid (Addgene #90331) into pcDNA4/His B vector with Flag-tag at N-terminal (pcDNA4-Flag-BRD4-WT). Point-mutations (BRD4-Y97A, Y98A, Y97/98A, Y399A, and Y590A) were introduced with PCR method using the primers with the desired mutations which alter the tyrosine 97, 98, 399, or 590 residues to alanine, rendering the phosphorylation deficiency of BRD4 (Y97A, Y98A, Y97/98A, Y399A, and Y590A). Same strategy was used for construction of phosphorylation mimic of BRD4 (BRD4-Y97/98E).

For generation of the WT or constitutively active mutant JAK2 overexpressing plasmid, the full-length *JAK2* DNA were cut from pDONR223-JAK2 plasmid (Addgene #23915) or JAK2 (V617F)-pcw107-V5 plasmid (Addgene #64610), and then inserted into pcDNA4/myc-His B vector with fusion myc-tag at C-terminal (pcDNA4-myc-JAK2-WT or pcDNA4-myc-JAK2-V617F). QuikChange Multi Site-Directed Mutagenesis Kit was used to alter the lysine 882 residue to arginine, rendering the kinase activity of JAK2 inactive (pcDNA4-myc-JAK2-K882R). Primer sequences for plasmids construction are shown in Supplementary Table 2.

Plasmid transfections were conducted using jetPRIME (Polyplus-transfection, New York, NY) or Lipofectamine 2000 (Invitrogen), according to the manufacturer’s instructions.

Cells were transfected with small interfering RNAs (siRNAs) using the Lipofectamine RNAiMAX (Invitrogen) following the manufacturer’s instructions All siRNAs were purchased from Sigma-Aldrich. The targeted sequences of siRNAs were listed in Supplementary Table 3.

### DUB Scan assay

To get ubiquitnated BRD4, flag-tagged BRD4 and ubiquitin were transfected into 293T cells. Cells were treated with MG132 for 12 h after 30 h transfection. Then cells were harvested and washed with cold PBS and lysed in RIPA buffer for 1 h. Insoluble cell debris were removed by centrifugation (16,000 × *g*, 15 min, 4 °C). cell lysate then was incubated with flag beads (sigma, #A2220) overnight with continuous stirring. Flag beads complex was then washed thoroughly thrice with washing buffer before flag-BRD4 was eluted with 100 μg/ml flag peptide (Sigma #F3290) in TBS at 4 °C. The concentration of BRD4 was determined by Bio-Rad Protein Assay Kit (Bio-Rad #5000006).

To identify the deubiquitinases (DUBs) responsible for BRD4 deubiquitylation, DUB Scan assay was performed using DUBscan™ Kit (UBQUIGENT, Cat. No. 67-0006-001) according to the manufactory’s instruction. In total, 1 μg purified BRD4 was added to each well of the DUBscan plate which was incubated on a plate shaker for 1 h at 37 °C. Samples were then denatured with Blue Loading Buffer (Cell Signaling Technology #7722) at 95 °C for 5 min. Western blotting analysis was performed with 4–20% SDS-PAGE as mentioned above. Anti-ubiquitin antibody was used for detection of ubiquitination of BRD4. And quantification of the density of BRD4 ubiquitination was determined by imagelab (Bio-Rad).

### In vivo experiments

All animal experiments were approved by the ASTAR-Biopolis Institutional Animal Care and Use Committee (IACUC) at Genome Institute of Singapore or the sixth affiliated hospital of Sun Yat-sen University. Six to eight-week-old NOD/MrkBomTac-Prkdcscid (NOD/SCID) or nude mice were purchased from InVivos (Singapore) or Gempharmatech Co., Ltd. (China). For PDC1, HT-29, and SW480 xenograft experiments, 5 × 10^5^ cancer cells were mixed and resuspended in 1:1 of Matrigel (BD, catalog number: #354230) in 100 µL total volume, then injected subcutaneously into flanks of mice. For co-injection experiments, 5 × 10^5^ cancer cells and 5 × 10^5^ CAF cells were mixed and grafted into the mice. Tumor measurement started when tumors became palpable and was performed twice a week using Vernier caliper. Tumor volume was calculated with the following formula: *V* = W × W × L/2. Randomization was performed by equally dividing tumor-bearing mice of similar tumor burden into control and experimental groups for drug treatment. No experimental samples were excluded in this study, with the exception of animals that died from surgery or unexpected illness.

For drug treatment experiments, the treatment began when the average of xenograft tumor volume reached 100 mm^3^. 100 mg/kg (+)-JQ1 (10% DMSO + 10% Cremophor + 10% ethanol + 70% saline) was given by intraperitoneal injection daily. In total, 50 mg/kg dBET1(10% DMSO + 20% Cremophor + 20% Ethanol + 50% Saline) was given by intraperitoneal injection daily. In all, 75 mg/kg Reparixin (10% DMSO + 40% PEG300 + 5% Tween-80 + 45% Saline), 50 mg/kg Tocilizumab were given by intraperitoneal injection twice per three days. In total, 150 mg/kg Pacritinib (10%DMSO + 0.5% methylcellulose and 0.1% Tween-80) was given by oral gavage daily. After 2–4 weeks of treatment, the mice were euthanized and tumors were collected for further analysis.

### Tissue specimens and IHC staining

Human colorectal cancer tissue microarrays (TMAs) for immunochemistry (IHC) analysis and resected colorectal cancer tissue specimen were obtained from The Sixth Affiliated Hospital, Sun Yat-sen University (China) as described previously^47^. Studies with these samples were approved by the Ethics Committee of the Sixth Affiliated Hospital, Sun Yat-sen University (China). Informed written consent had been previously obtained from each individual who agreed to provide tissue for research purposes. The commercial human colorectal cancer TMA was purchased from Pantomics Inc. (Richmond, CA; catalog number: #COM961), which included 36 paired primary and metastatic tumor tissues.

For TMAs, 200 and 66 consecutive patients with high-risk stage II or III colorectal adenocarcinoma treated between May 2007 and May 2012 were included in the study. All patients underwent quality assessed curative surgery and had received at least 3-month oxaliplatin based adjuvant chemotherapy in The Sixth Affiliated Hospital of Sun Yat-sen University^47^.

IHC and image analyses of TMAs were conducted by Histopathology Department of the Institute of Molecular and Cell Biology (IMCB), A*STAR, Singapore. Briefly, TMAs were deparaffinized, rehydrated, and antigens were retrieved by sodium citrate (pH 6) for 40 min at 100 °C; sections were then incubated in 3–4% H_2_O_2_ for 15 min at room temperature to block endogenous peroxidase. Slides were incubated with BRD4 antibody from Bethyl Laboratories (Montgomery #A301-985A100, 1:2500), phospho-Y1007/Y1008-JAK2 from Abcam (#ab32101, 1:250 dilution), α-SMA antibody from Sigma (#A2547, 1:7500), or pBRD4 antibody (1:1000) overnight at 4 °C, followed by 5 min incubation with anti-mouse/rabbit Labelled Polymer (Leica Biosystems). The detection system was DAB-based Bond™ Polymer Refine Detection (Leica Biosystems). Slides were scanned at ×20 using a Leica SCN400 slide scanner (Leica Microsystems). Images were exported to Slidepath Digital Image Hub (Leica Microsystems) for viewing. TMA cores were analyzed using the Measure Stained Cells algorithm of Slidepath Tissue IA software (Leica Microsystems, 3.0). H-score method was applied to semi-quantified the IHC staining of individual protein using the following formula: [(% cell at 1 + ) × 1 + (% cell at 2 + ) × 2 + (% cell at 3 + ) × 3], where the 1+, 2+, and 3+ stand for the weak, moderate, and strong intensity, respectively, and the % is the percentage of the cells with that intensity. H-score generated a continuous variable ranging from 0 to 300. Scanning and image analysis was performed by the Advanced Molecular Pathology Laboratory, IMCB, Singapore. H-score of individual protein was then transformed to *z*-score value by the formula: *Z* = $\frac{X-\mu }{\sigma }$ (where *X* stands for H-score value, *µ* stands for mean, and *σ* stands for standard deviation). The cut-off of *z*-score was set as “Median” to determine the high or low expression of protein of interest. The disease-free survival curve was plotted using Kaplan–Meier method, and the statistical parameters were calculated by log-rank (Mantel–Cox) test using GraphPad Prism software (Version 7.0).

### Three-dimensional cell culture

The three-dimensional (3D) cell culture methods were adapted from the previously described procedures^48^. In brief, the eight-well chamber slides (Falcon #354656) were pre-coated with 7.6 mg/ml growth factor-reduced Matrigel (Falcon #354230) for 30 min at 37 °C. PDC1 cells were trypsinized and suspended in DMEM medium containing 10% FBS and 150 µg/ml Matrigel. Approximately 5 × 10^3^ cells were seeded in each well. Every third day, the cells were replenished with fresh DMEM containing 10% fetal bovine serum. And cell growth was monitored by imaging.

### Transwell invasion and migration assay

Transwell invasion/migration assay was performed using 24-well FluoroBlok transwell insert (Falcon #351158) with a pore size of 8 µm according to manufacturer’s instruction. For invasion assay, at day 1 the inserts were pre-coated with growth factor-reduced Matrigel (BD Biosciences #354230) at the concentration of 200 μg/ml for 2 hr at 37 ^o^C. Then 1 × 10^5^ of PDC1 cells were seeded into each insert in DMEM medium containing 10%FBS. In all, 4 × 10^4^ of CAFs were seeded into each well of 24-well plate which was pre-coated with collagen (Sigma #C8919) at day 2. Next day, the CAFs were replenished with fresh DMEM containing 10%FBS, and PDC1 cells were carefully replenished with low serum (0.25%) DMEM medium. Then the inserts were subsequently immersed into the wells with CAFs or medium only (for non-coculture control). For drug treatment, 2.5 μg/ml Tocilizumab or/and 1 μM Reparixin were added into the medium at day 3. Invaded cells were fixed after 72 h of incubation by using 3.7% formaldehyde and stained with crystal violet. Then the non-invaded cells were carefully removed with cotton sticks. The invaded cells were photographed and counted with ImageJ. To determine the migration ability of the cancer cells, similar procedures were performed with the exception that the PDC1 cells were directly seeded into the inserts without Matrigel coated.

### ChIP and ChIP-seq Assays

Chromatin immunoprecipitation (ChIP) was carried out with minor modifications of a previous procedure^44^. Briefly, 1 million cells (per sample) were cross-linked using 1% formaldehyde for 10 min and reactions were stopped by adding 0.125 M glycine for 5 min at room temperature. Cell pellets were washed with cold TBSE buffer (20 mM Tris-HCl pH 7.5, 150 mM NaCl, 1 mM EDTA) for three times. Then cells were lysed in SDS lysis buffer (50 mM Tris-HCl pH8.0, 10 mM EDTA, 1% SDS) for 5 min on ice. Sonication conditions were optimized for tumor cells using Branson Digital Sonifier to achieve a shear length of 250–500 bp. Solubilized chromatin was diluted 10 times in dilution buffer (10 mM Tris-HCl pH 7.4, 140 mM NaCl, 1 mM EDTA, 1% Triton X-100, 0.01% SDS) and precleared with Dynabead (Life Technologies) for 4 h at 4 °C. Precleared chromatin was then incubated with appropriate antibodies overnight at 4 °C. The following primary antibodies were used for Chromatin-IP: pY97/98-BRD4 (homemade, iDNA), BRD4 (Cell Signaling, Cat#13440, RRID:AB_2687578), Phospho-Stat3 (Tyr705) (Cell Signaling, Cat#9145, RRID:AB_2491009), and H3K27Ac (Abcam, Cat#ab4729, RRID:AB_2118291). Then the complexes of chromatin and antibodies were captured with Dynabead at 4 °C for 2 h. Immune complexes were then washed thrice with dilution buffer and once with TE buffer (10 mM Tris-HCl pH 7.5, 1 mM EDTA), sequentially. Elution and reverse-crosslinking were performed in elution buffer (50 mM Tris-HCl pH 7.5, 10 mM EDTA, 1% SDS) at 68 °C with vortex overnight followed by digestion with proteinase K (20 mg/ml) for 2 h at 42 °C. Eluted DNA was purified using Phenol-CHCl_3_-Isoamylalchol and then precipitated with ethanol with existence of NaCl and yeast tRNA. DNA was resuspended in water with RNase A and incubated in 37 °C for 10 min.

For ChIP-Seq, precipitated chromatin DNA was sent to Novogene (China) for library preparation and sequenced in HiSeq2500 (Illumina). ChIP-seq data were generated using vehicle and rIL6/8 treated samples. Briefly clean reads were aligned to reference genome hg38 by Bowtie2(v2.4.1)^49^. Unmapped reads and PCR duplicates were removed using samtools (v1.7)^50^ and Picard (v2.23.3) (http://broadinstitute.github.io/picard). RPKM normalized bigwigs were generated by bamCoverage command from deepTools (v3.4.3)^51^. Narrow peaks were called by MACS2 (v2.2.7.1)^52^ using input sequences as a background control. Peaks were annotated to gene features using HOMER (v4.11)^53^. Scores for each genome regions were calculate using deepTools and visualized using matplotlib(v3.2.2)^54^ package for python3. Peaks were visualized using Integrative Genomics Viewer^55^. For SE analysis, bam files from H3K27ac ChIP-seq datasets were used. HOMER was used for SEs identification. ChIPseeker R package^56^ was used to annotate the peaks with nearest gene, followed by the identification of common genes in samples. As a result, 176 common genes found in treated pBRD4, pSTAT3, and H3K27ac samples.

### Statistical analysis

All in vitro experiments were repeated at least two times in technical triplicates unless stated otherwise. All data are analyzed with GraphPad Prism version 7 or 8 (GraphPad Software; San Diego, CA) and expressed as means ± s.e.m. Statistical significance was calculated using two-tailed Student’s *t* test, unless stated otherwise. *P*-values of <0.05 (typically ≤ 0.05) were regarded as statistically significant. NS stands for not statistically significant.

### Reporting summary

Further information on research design is available in the Nature Research Reporting Summary linked to this article.

## Supplementary information

Supplementary Information

Reporting summary

## Data Availability

The microarray data and ChIP-Seq data generated in this study have been deposited in the Gene Expression Omnibus (GEO) under accession numbers GSE160035 and GSE160352 respectively. The online dataset GSE39396 used in this study is available in GEO dataset. Source data are provided with this paper.

## Source data

Source data file
