# Supplementary material for: Stromal induction of BRD4 phosphorylation Results in Chromatin Remodeling and BET inhibitor Resistance in Colorectal Cancer
Source: Nat Commun. 2021 Jul 21;12:4441. doi: 10.1038/s41467-021-24687-4 (PMC8295257; doi:10.1038/s41467-021-24687-4)
Supplement: Supplementary file 1 — Supplementary Information [file 41467_2021_24687_MOESM1_ESM.pdf]

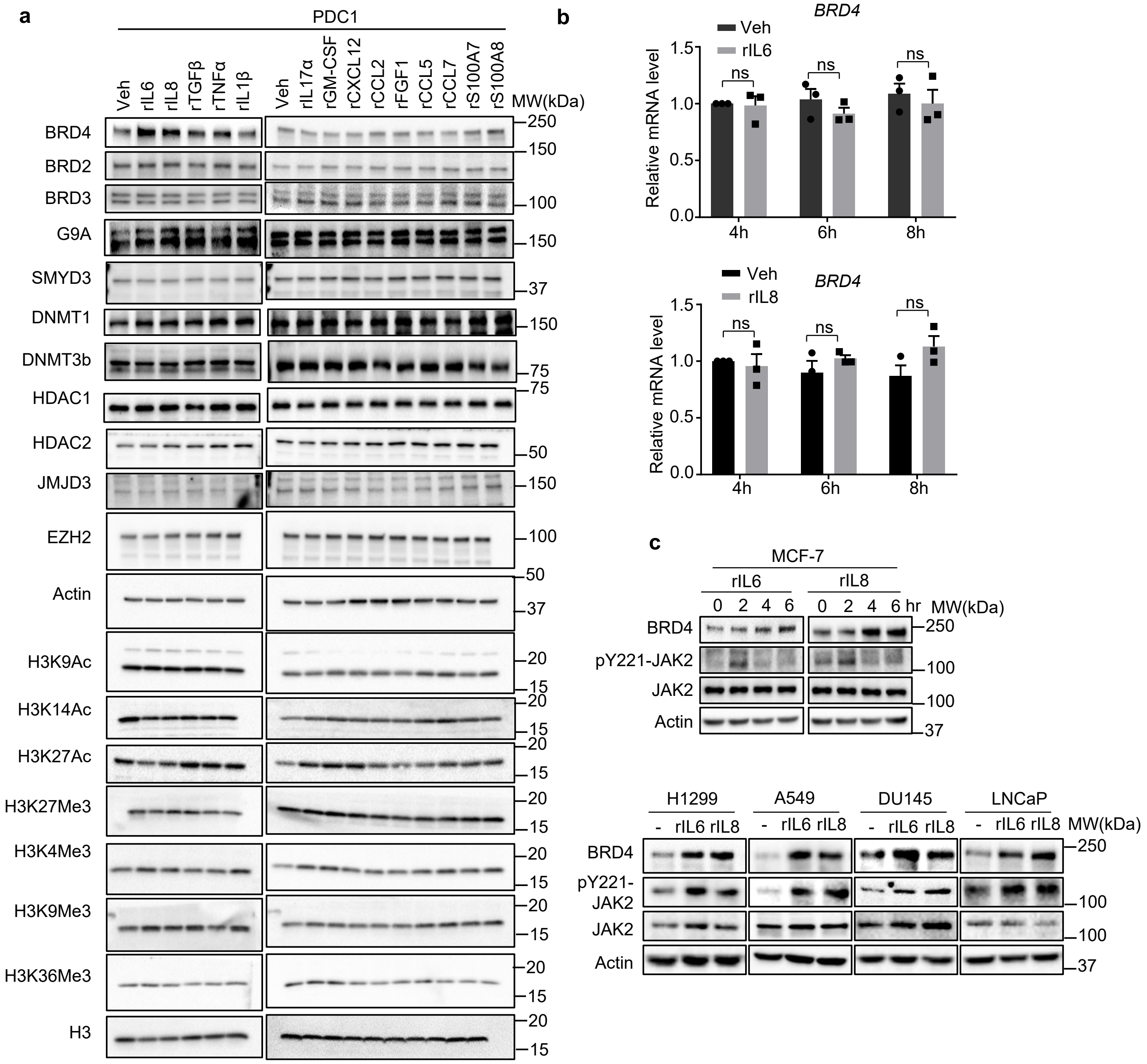

Supplementary Figure 1. IL6 and IL8 induce BRD4 protein expression.

(**a**)Representative western blot analysis (n=2) of indicated proteins in PDC1 cells after treatment with a panel of 14 inflammatory cytokines. (**b**)qRT-PCR analysis of BRD4 expression in PDC1 cells in Figure 1b. (**c**)Representative western blot analysis (n=2) of indicated proteins in lung cancer cells (H1299, A549), prostate cancer cells (DU145, LNCaP) and breast cell lines (MCF7, MCF10A) were treated with recombinant IL6 or IL8. In (**b**), data is presented as the mean ± SEM (error bars) of three biological replicates. The *p* values were determined using two-tailed Student's t tests. ns indicates non-significant.

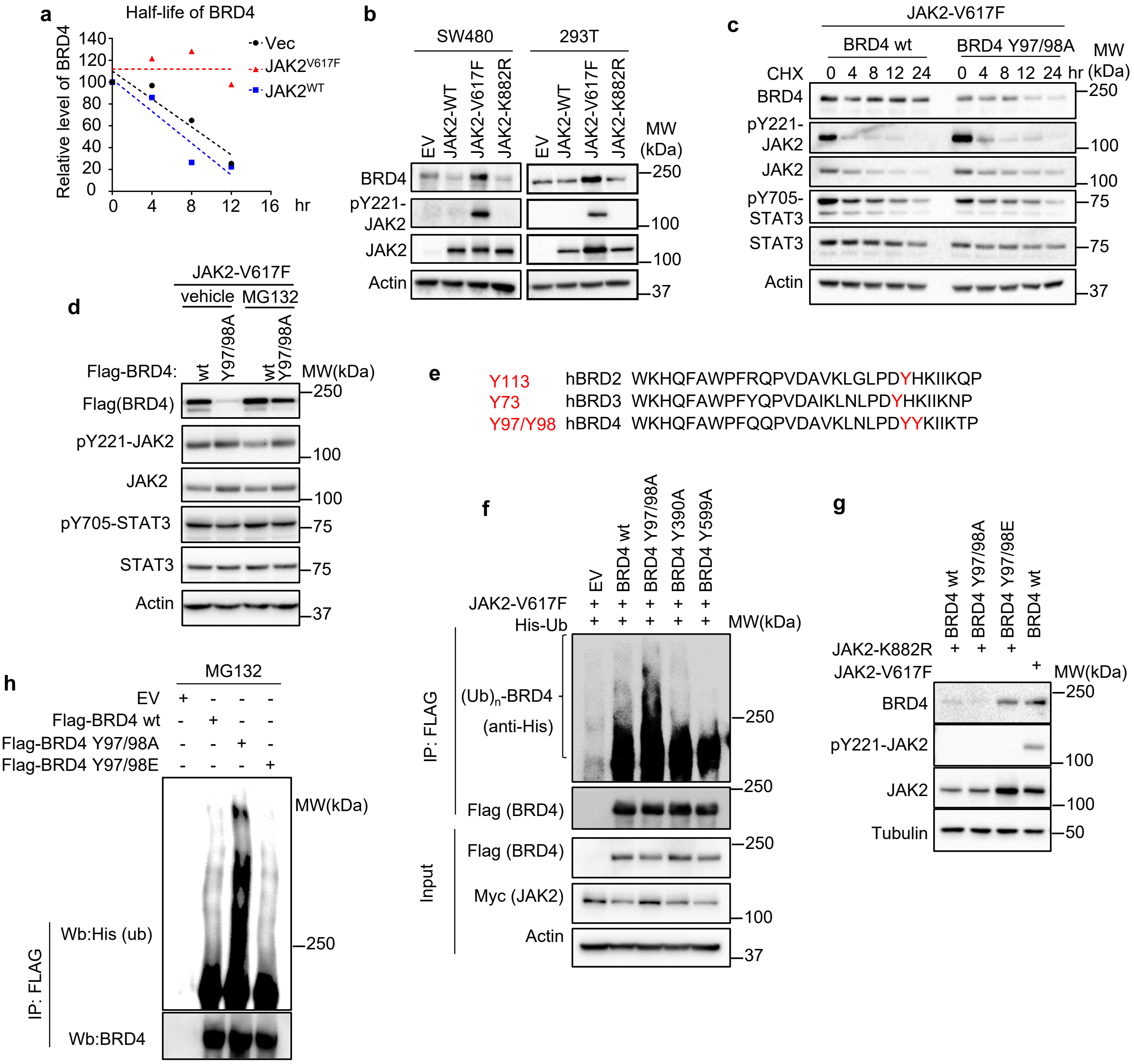

Supplementary Figure 2. JAK2-induced stabilization of BRD4 depends on tyrosine 97/98 of BRD4.
(**a**)Quantification of BRD4 protein level in Figure 2a. (**b**) Representative western blot analysis (n=3) of indicated proteins in SW480, 293T cells transfected with indicated constructs. (**c**) Representative western blot analysis (n=2) of indicated proteins in 293T cells transfected with BRD4-wildtype or BRD4-Y97/98A together with active JAK2-V617F. Cells were treated with 50μg/ml CHX and harvested at different time point. (**d**) Representative western blot analysis (n=2) of indicated proteins in 293T cells transfected with BRD4-wildtype or BRD4-Y97/98A together with active JAK2-V617F in the presence of 20μM MG132 or vehicle. (**e**) Amino acid sequence alignment of putative phosphorylation motifs in human BRD2, BRD3 and BRD4. Predicted phosphorylated tyrosine was depicted in red. (**f**) In vitro ubiquitination assays (n=3) of WCL and immunoprecipitates from 293T cells transfected with indicated constructs in the presence of 20μM MG132. (**g**) Representative western blot analysis (n=2) of indicated proteins in 293T cells transfected with BRD4 mutants together with inactive or active JAK2-V617F. (**h**) In vitro ubiquitination assays (n=2) of WCL and immunoprecipitates from 293T cells transfected with indicated constructs together with active JAK2-V617F in the presence of 20μM MG132.

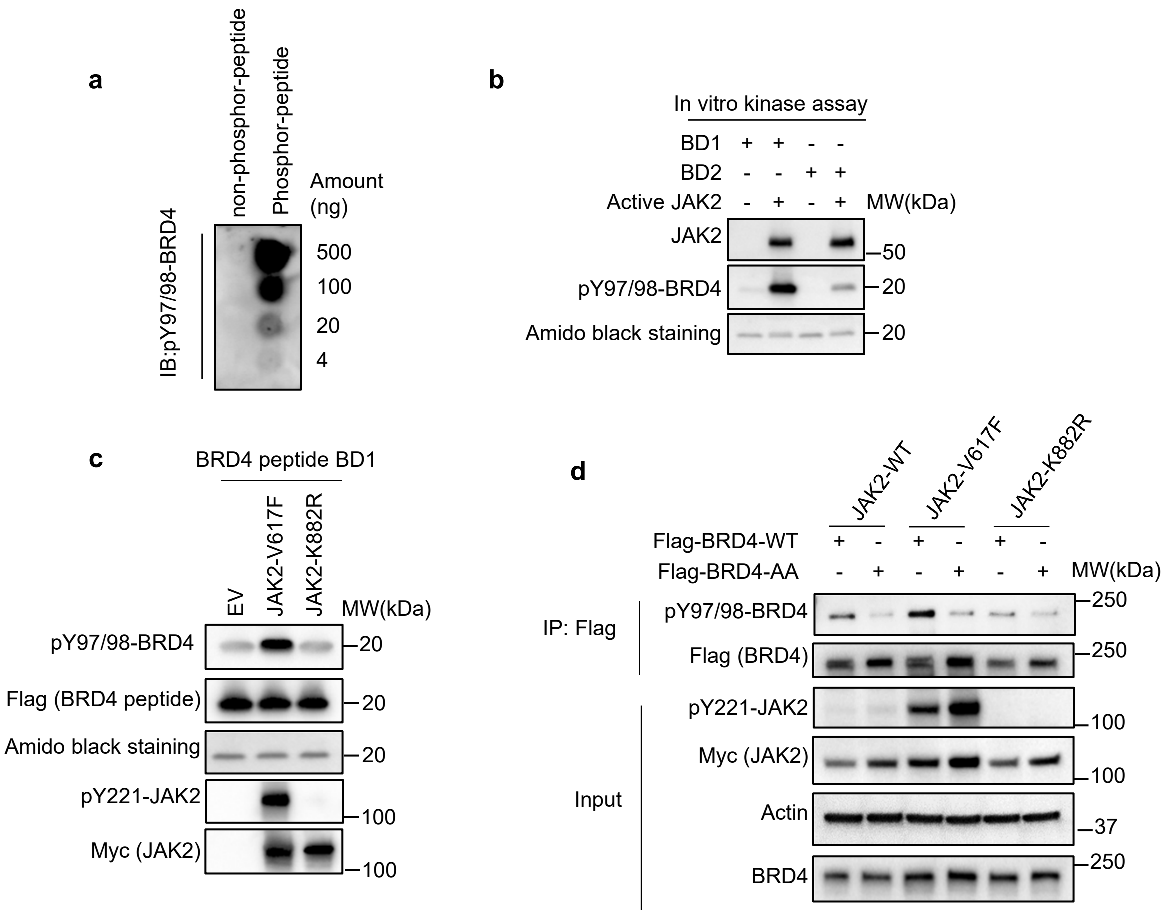

Supplementary Figure 3. JAK2 induces tyrosine phosphorylation of BRD4 at Y97/Y98 .

(a) Anti-phosphorylated-BRD4 antibody specificity was tested by dot-blot analysis (n=4). **(b)** Representative western blot analysis (n=3) of BRD4 peptides (BD1 and BD2) from kinase assay co-incubated with active JAK2-V617F. **(c)** Representative western blot analysis (n=3) of BRD4 peptide (BD1) from kinase assay co-incubated with JAK2-V617F or JAK2-K882R. **(d)** Representative western blot analysis (n=2) of WCL and immunoprecipitates by anti-Flag antibody from 293T cells transfected with indicated constructs and treated with 20μM MG132.

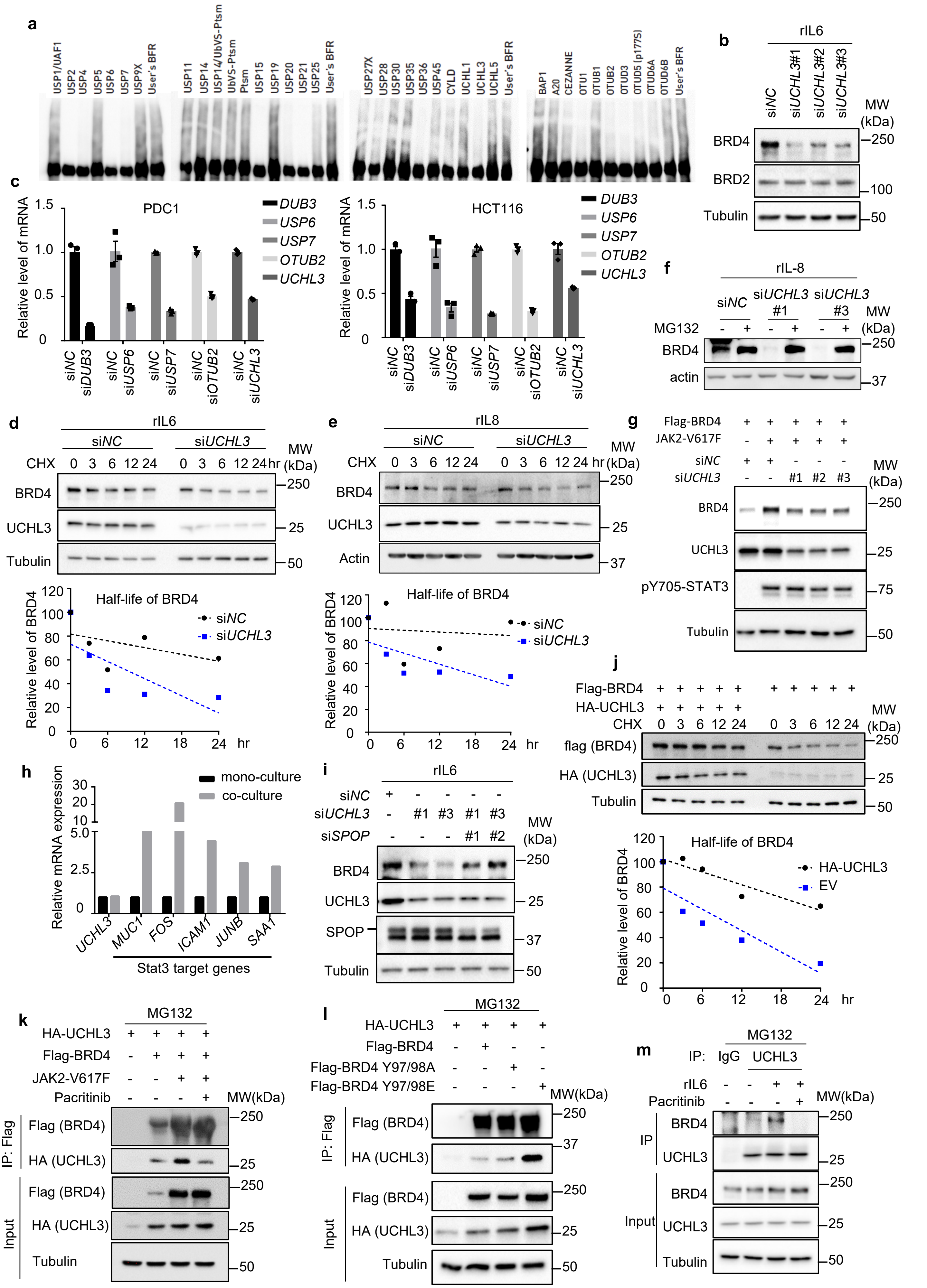
a
USP1/UAF1
USP2
USP4
USP5
USP6
USP7
USP9X
User's BFR
USP11
USP14
USP14/UbVS-Ptsm
UbVS-Ptsm
Ptsm
USP15
USP19
USP20
USP21
USP25
User's BFR
USP27X
USP28
USP30
USP35
USP36
USP45
CYLD
UCHL1
UCHL3
UCHL5
User's BFR
BAP1
A20
CEZANNE
OTU1
OTUB1
OTUB2
OTUD3
OTUD5 (p177S)
OTUD6A
OTUD6B
User's BFR
b
rIL6
siNC
siUCHL3#1
siUCHL3#2
siUCHL3#3
MW (kDa)
BRD4
BRD2
Tubulin
250
100
50
c
PDC1
HCT116
Relative level of mRNA
DUB3
USP6
USP7
OTUB2
UCHL3
siNC
siDUB3
siUSP6
siUSP7
siOTUB2
siUCHL3
f
rIL-8
siNC
siUCHL3 #1
siUCHL3 #3
MG132
BRD4
actin
250
37
d
rIL6
siNC
siUCHL3
CHX 0 3 6 12 24 hr
BRD4
UCHL3
Tubulin
Half-life of BRD4
Relative level of BRD4
e
rIL8
Actin
g
Flag-BRD4
JAK2-V617F
siNC
siUCHL3
#1 #2 #3
pY705-STAT3
75
h
mono-culture
co-culture
Relative mRNA expression
UCHL3
MUC1
FOS
ICAM1
JUNB
SAA1
Stat3 target genes
i
siSPOP
SPOP
j
HA-UCHL3
flag (BRD4)
HA (UCHL3)
EV
k
MG132
Pacritinib
IP: Flag
Flag (BRD4)
Input
MW(kDa)
l
Flag-BRD4 Y97/98A
Flag-BRD4 Y97/98E
m
IP: IgG UCHL3
rIL6
IP

Supplementary Figure 4. UCHL3 promotes BRD4 stabilization.

(a) Representative western blot analysis (n=1) of ubiquitinated BRD4 from DUB Scan assay. **(b)** Representative western blot analysis (n=2) of indicated proteins in HCT116 cells transfected with siUCHL3 oligos in the presence of 50ng/ml rIL6. **(c)** Q-PCR analysis of mRNA level of indicated gene in PDC1 or HCT116 in Figure 3c transfected with indicated siRNA oligos. Data are expressed as mean ± SEM of three independent experiments. **(d)** and **(e)**Top: Representative western blot analysis (n=2) of indicated proteins in PDC1 cells transfected with indicated siRNA oligos, and treated with 50ng/ml rIL6 (c), 50ng/ml rIL8 (d) or 50μg/ml CHX as indicated. Bottom: Quantification of BRD4 protein level. **(f)** Representative western blot analysis (n=1) of indicated proteins in PDC1 cells transfected with indicated siRNAs with/without 20μg/ml MG132 under rIL8 (50ng/ml) treatment. **(g)** Representative western blot analysis (n=2) of indicated proteins in 293T cells transfected with indicated constructs or siRNAs. **(h)** Relative mRNA level of UCHL3 and some STAT3 target genes in RNA-Seq data in PDC1 cells which were mono-cultured or co-cultured with CAF1. **(i)** Representative western blot analysis (n=2) of indicated proteins in PDC1 cells transfected with siUCHL3 and/or siSPOP oligos in the presence of 50ng/ml rIL6. **(j)** Top: Representative western blot analysis (n=1) of indicated proteins in 293T cells transfected with indicated constructs, then treated with 50μg/ml CHX as indicated. Bottom: Quantification of BRD4 protein level. **(k)** Representative western blot analysis (n=2) of WCL and immunoprecipitates by anti-Flag antibody from 293T cells transfected with indicated constructs and treated with 20μM MG132 or 2.5μM pacritinib as indicated. **(l)** Representative western blot analysis (n=2) of WCL and immunoprecipitates by anti-Flag antibody from 293T cells transfected with indicated constructs and treated with 20μM MG132. **(m)** Representative western blot analysis (n=2) of WCL and immunoprecipitates by anti-UCHL3 antibody from PDC1 cells treated with 50ng/ml rIL6 and/or 2.5μM pacritinib as indicated.

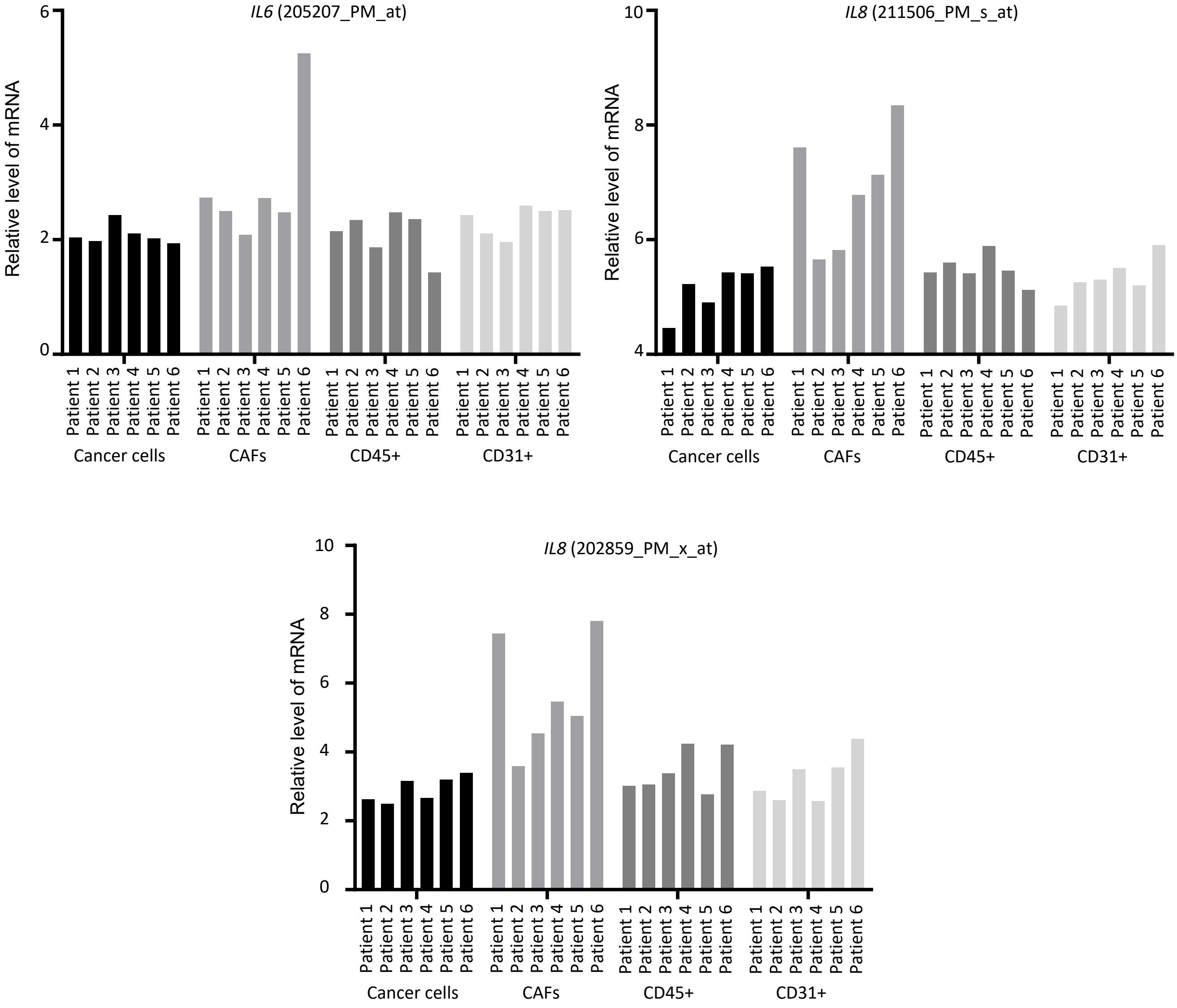

Supplementary Figure 5. CAF expresses high level of IL6/IL8 in tumor.
Expression analysis of IL6/IL8 in CAF, endothelial cells, cancer cells and immune cells in CRC patients in published dataset GSE39396.

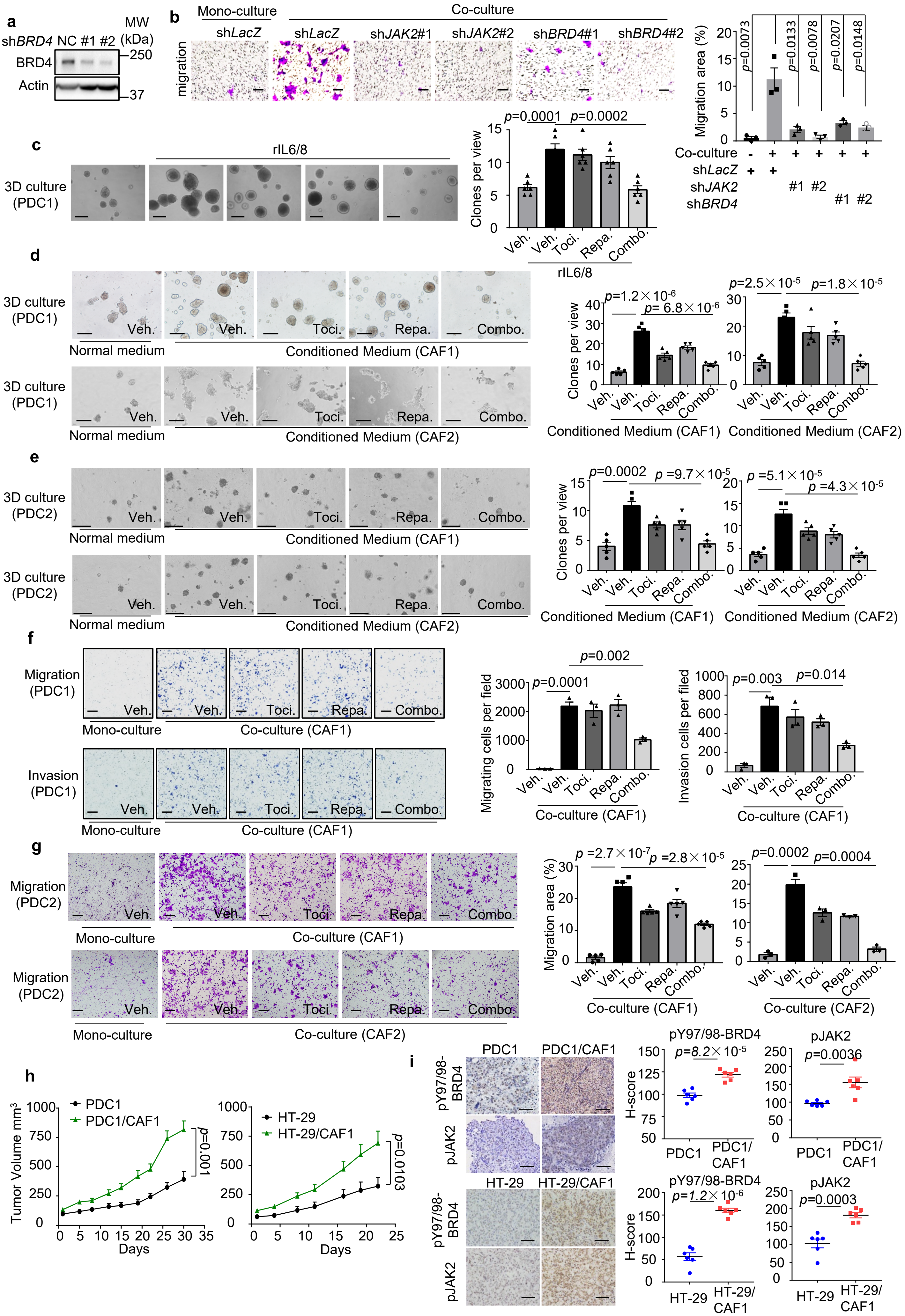

a
shBRD4 NC #1 #2
MW (kDa)
BRD4
Actin
250
37
b
Mono-culture
Co-culture
shLacZ
shLacZ
shJAK2#1
shJAK2#2
shBRD4#1
shBRD4#2
migration
Migration area (%)
p=0.0073
p=0.0133
p=0.0078
p=0.0207
p=0.0148
Co-culture
shLacZ
shJAK2
shBRD4
c
3D culture (PDC1)
rIL6/8
Clones per view
p=0.0001
p=0.0002
Veh.
Toci.
Repa.
Combo.
d
Normal medium
Conditioned Medium (CAF1)
Conditioned Medium (CAF2)
p=1.2×10-6
p= 6.8×10-6
p=2.5×10-5
p=1.8×10-5
e
3D culture (PDC2)
p=0.0002
p =9.7×10-5
p =5.1×10-5
p =4.3×10-5
f
Migration (PDC1)
Invasion (PDC1)
Mono-culture
Co-culture (CAF1)
Migrating cells per field
Invasion cells per filed
p=0.002
p=0.0001
p=0.003
p=0.014
g
Migration (PDC2)
Co-culture (CAF2)
p =2.7×10-7
p =2.8×10-5
p=0.0002
p=0.0004
h
Tumor Volume mm3
PDC1
PDC1/CAF1
p=0.001
HT-29
HT-29/CAF1
p=0.0103
Days
i
PDC1
PDC1/CAF1
HT-29
HT-29/CAF1
pY97/98-BRD4
pJAK2
H-score
p=8.2×10-5
p=0.0036
p=1.2×10-6
p=0.0003

Supplementary Figure 6. Cancer-associated fibroblasts promotes migration, invasion and tumor growth of cancer cells through JAK2/pBRD4.

(a) Representative western blot analysis (n=3) of BRD4 in PDC1 cells transfected with indicated siRNAs. **(b)** Representative images (Left panel) and quantification (Right panel, n=3) of migration cells PDC1 cells expressing indicated Tet-on-shRNAs were mono-cultured or cocultured with CAF with 100ng/ml doxycycline (Scale bars, 50 μm). **(c)** Representative images (Left panel) and quantification (Right panel, 3 independent experiments performed in duplicate) of cell growth in 3D Matrigel. PDC1 cells were cultured on Matrigel supplied with/without 50ng/ml rIL6/8. 2.5μM Tocilizumab, 1μM reparixin or combination were used as indicated to block the receptors of IL6 and IL8 (Scale bars, 100 μm). **(d)** and **(e)** Representative images (Left panel) and quantification (Right panel, 2 experiments with 5 replicates) of cell growth in 3D Matrigel. PDC1 cells (d) or PDC2 cells (e) were mono-cultured or cocultured with CAF1 or CAF2 supplied with 2.5μM Tocilizumab, 1μM reparixin or combination (Scale bars, **d**,100 μm; **e**, 50 μm). **(f)** Representative images (Left panel) and quantification (Right panel, n=3) of migratory or invasive cells. PDC1 cells were mono-cultured or cocultured with CAF1 supplied with 2.5μM Tocilizumab, 1μM reparixin or combination (Scale bars, 100 μm). **(g)** Representative images (Left panel) and quantification (Right panel, 2 experiments with 5 (with CAF1) or 3 replicates (with CAF2)) of migratory cells. PDC2 cells were mono-cultured or cocultured with CAF1 or CAF2 supplied with 2.5μM Tocilizumab, 1μM reparixin or combination (Scale bars, 100 μm). **(h)** Immuno-deficient nude mice were engrafted with PDC1 or HT-29 cells together with/without CAF1 cells. Tumor volume was measured twice a week. PDC1(n=7), PDC1/CAF1(n=8), HT-29(n=9), and HT-29/CAF1(n=9). **(i)** Representative IHC staining (Left panel) and quantification (Right panel, n=6 independent xenografts samples from each group) for pJAK2, pY97/98-BRD4 in PDC1 or HT-29 xenografts. (Scale bars, 20 μm).

Data is presented as the mean ± SEM (error bars) of three independent experiments unless stated otherwise. The p values were determined using two-tailed Student's t tests.

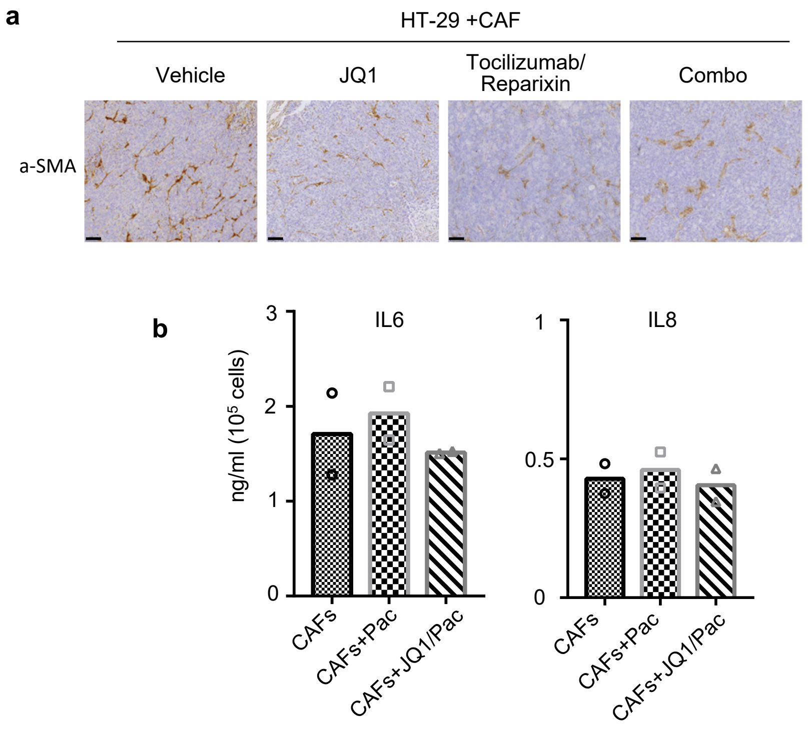

Supplementary Figure 7. Analysis of CAFs presence and IL6/8 secetion by CAFs after treatment.

(a) Representative IHC staining from three independent HT-29 xenografts for α-SMA in Figure 5F (Scale bars, 100 μm). **(b)** ELISA analysis of indicated cytokines (n=2) in the culture medium where CAF1 cells were treated with indicated compounds.

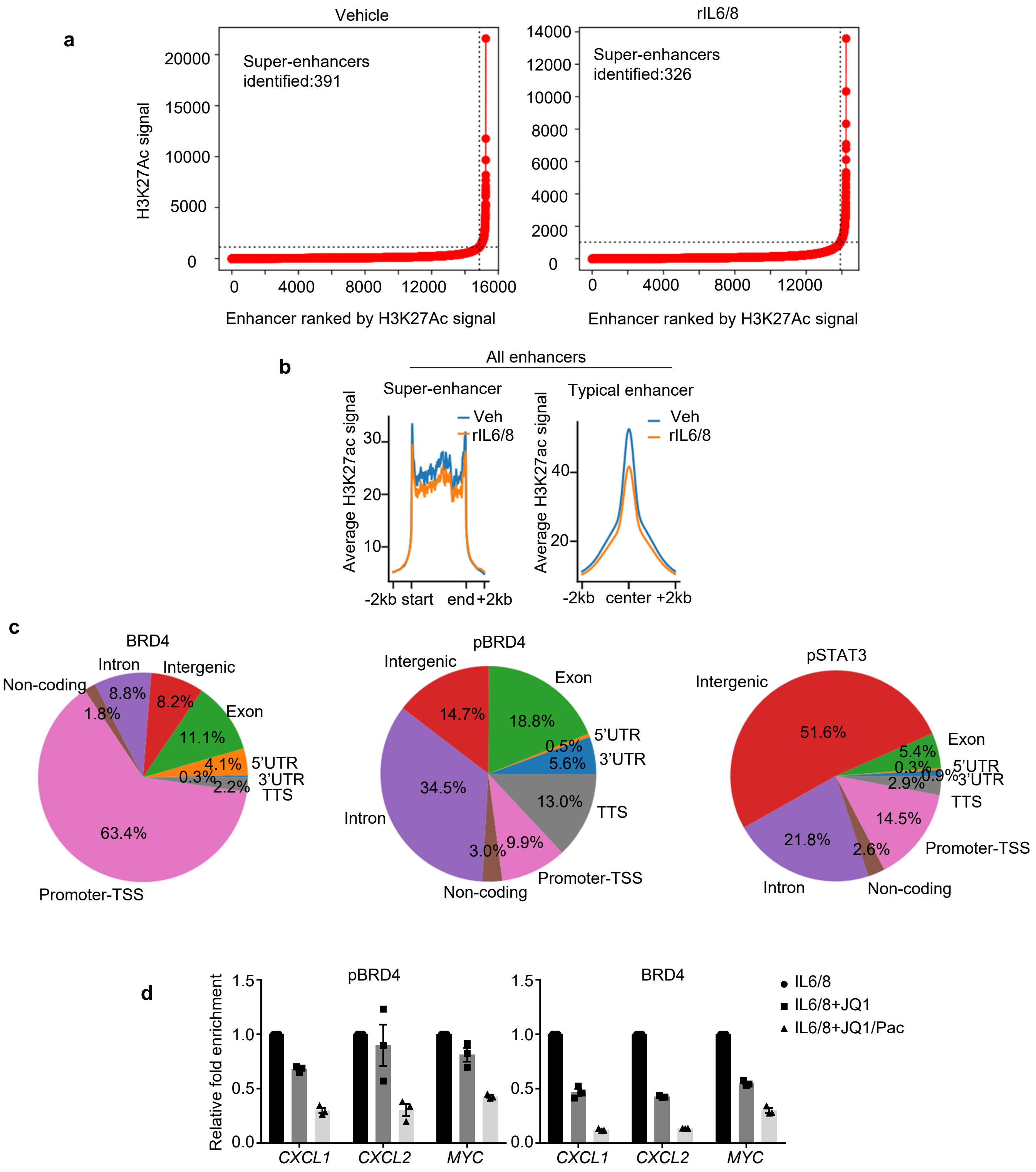

Supplementary Figure 8. Chromatin binding analysis in PDC1 cells under treatment.

(a) Plot indicating super-enhancers identified on the basis of H3K27ac signals in vehicle or rIL6/8 treated PDC1 cells. **(b)** Average intensity curves of ChIP-seq reads for H3K27ac at super-enhancer (Left panel) and typical enhancer (Right panel) before and after rIL6/8 treatment based on ChIP-seq in PDC1 cells. **(c)** Pie chart showing the distribution of BRD4, pBRD4 and pSTAT3 peaks across gene features (Promoters, TSS, 5'UTR, 3'UTR, exons, introns, non-coding and intergenic regions). **(d)** ChIP-qPCR analysis (n=3) of BRD4 and pY97/98-BRD4 enrichments on *CXCL1*, *CXCL2* and *C-MYC* promoters in the PDC1 cells which were treated with indicated chemicals. Data are expressed as mean ± SEM of three independent experiments.

Supplementary Table 1. List of gene-specific primers used for quantitative RT-PCR.

Gene	Forward or reverse	Sequence
ACTB	F	CCCCGCGAGCACAGAG
	R	TCATCATCCATGGTGAGCTGG
BRD4	F	CAACAAGCCTGGAGATGACA
	R	TGTTTCTTTCCTCCCACG
GAPDH	F	TGCACCACCAACTGCTTAGC
	R	GGCATGGACTGTGGTCATGAG
CXCL1	F	AGCTTGCCTCAATCCTGCATCC
	R	TCCTTCAGGAACAGCCACCAGT
CXCL2	F	GGCAGAAAGCTTGTCTCAACCC
	R	CTCCTTCAGGAACAGCCACCAA
BMP4	F	CTGGTCTTGAGTATCCTGAGCG
	R	TCACCTCGTTCTCAGGGATGCT
MYC	F	CAGCTGCTTAGACGCTGGATT
	R	GTAGAAATACGGCTGCACCGA
FAT1	F	ATCTGTGGAGCCTCCTGGCATA
	R	CATCTGTAGCCTCGACTGTGAG

Supplementary Table 2. List of primers used for cloning.

Primers	Forward or reverse	Sequence
UCHL3 from pDEST-LTR into pcDNA4/His/B	F	TGGAATTCTATGTACCCATACGATGTTCCAG
	R	AGCGGCCGCCGTATGCTGCAGAAAGAGC
BRD4 from pcDNA5 into pcDNA4/His/B	F	ACTTAAGCTTCCACCATGGATTACAAGG
	R	CTGACTCGAGTCAGAAAAGATTTTCTTCAAATATTG
For BRD4-Y599A	F	agccccctcccacggctgagtcggaggaag
	R	cttcctccgactcagccgtgggagggggct
For BRD4-Y390A	F	cactgggcctacacgacgcctgtgacatcatcaagc
	R	gcttgatgatgtcacaggcgtcgtgtaggcccagtg
For BRD4-Y97/98A	F	ccgtcaagctgaacctccctgatgccgctaagatcattaaaacgcctatgg
	R	ccataggcgttttaatgatcttagcggcatcagggaggttcagcttgacgg
For BRD4-Y97A	F	gtcaagctgaacctccctgatgcctataagatcattaaaacgcc
	R	ggcgttttaatgatcttataggcatcagggaggttcagcttgac
For BRD4-Y98A	F	caagctgaacctccctgattacgctaagatcattaaaacgcctatg
	R	cataggcgttttaatgatcttagcgtaatcagggaggttcagcttg
For BRD4-Y97/98E	F	tgccgtcaagctgaacctccctgatgaggagaagatcattaaaacgcctatg
	R	cataggcgttttaatgatcttctcctcatcagggaggttcagcttgacggca

Supplementary Table 3. List of siRNAs and shRNAs.

**shRNAs or siRNAs**	**Sequence**
sh*LacZ*	GTGACCAGCGAATACCTGT
sh*BRD4* #1	GCAGAACAAACCAAAGAAA
sh*BRD4* #2	GCCAAATGTCTACACAGTATA
sh*BRD4* #3	GCTCAAGACACTATGGAAA
sh*JAK2* #1	GCCCAATTTCGATGGATTT
sh*JAK2* #2	GCAGAATTAGCAAACCTTA
si*NC*	UAGCGACUAAACACAUCAA
si*USP6*	GCACAGUAGCAAACUCAUA
si*OTUB2*	CAGAGUGCCUCGGACCACA
si*USP7*	AGUCGUUCAGUCGUCGUAU
si*DUB3*	CGUACUUGUGAUUCAUCAA
si*UCHL3* #1	GGCACCAAGUAUAGAUGAG
si*UCHL3* #2	GGGACAAGAUGUUACAUCA
si*UCHL3* #3	GGCAAUUCGUUGAUGUAUA
si*SPOP* #1	GAUUCAAGAAAUUCAUCCGUA
si*SPOP* #2	CACAGAUCAAGGUAGUGAAAU
